# Plasmon-enhanced Quantitative Lateral Flow Assay for Femtomolar Detection of SARS-CoV-2 Antibodies and Antigens

**DOI:** 10.21203/rs.3.rs-1258688/v1

**Published:** 2022-02-21

**Authors:** Rohit Gupta, Prashant Gupta, Zheyu Wang, Anushree Seth, Jeremiah Morrissey, Ige George, Sumanth Gandra, Gregory Storch, Bijal Parikh, Guy Genin, Srikanth Singamaneni

**Affiliations:** Washington University in St. Louis; Washington University in St. Louis; Washington University in St. Louis; Washington University in St. Louis; Washington University in St. Louis; Washington University School of Medicine; Washington University School of Medicine; Washington University School of Medicine; Washington University in St. Louis; Washington University in St. Louis

**Keywords:** Lateral flow assay, point-of-care diagnostic, COVID-19, serological assay, SARS-CoV-2 antigens, nucleocapsid protein

## Abstract

Lateral flow assays (LFAs) are the cornerstone of point-of-care diagnostics. Although rapid and inexpensive, they are 1000-fold less sensitive than laboratory-based tests and cannot be used for definitive negative diagnosis. Here, we overcome this fundamental limitation by employing plasmonically-enhanced nanoscale colorimetric and fluorescent labels. Plasmonic LFAs (p-LFAs) enabled ultrasensitive detection and quantification of low abundance analytes, without compromising the direct visual detection of conventional LFAs. Dynamic ranges and limits of detection were up to 100-fold superior to “gold standard” ELISA (enzyme-linked immunosorbent assay). p-LFAs had sample-to-answer time of 20 min, compared to 4 hours for ELISA, while achieving over 95% analytical sensitivity and 100% analytical specificity for antibodies and antigens of SARS-CoV-2 in human specimens. We also demonstrate that the p-LFAs enable quantitative detection of the target analytes in a standard-free manner. p-LFAs offer potential as a broadly adaptable point-of-care diagnostic platform that outperforms standard laboratory tests in sensitivity, speed, dynamic range, ease of use, and cost.

## 1. Introduction

Lateral flow (immuno)assays (LFAs) are amongst the simplest, fastest, and cheapest point-of-care (POC) diagnostic platforms, and offer broad potential for population-level screening for disease.^1–2^ However, this potential has not yet been fully achieved. Although numerous LFAs for severe acute respiratory syndrome coronavirus 2 (SARS-CoV-2) antibodies^3–5^ and antigens^6–7^ have been introduced, none have sensitivity and quantitation comparable to laboratory-based diagnostics such as real-time reverse transcription polymerase chain reaction (RT-PCR) and enzyme-linked immunosorbent assay (ELISA), which constrains their widespread use.^8–10^ In general, conventional colorimetric-LFAs are ~1000-fold less sensitive than these standard laboratory tests,^11–12^ and diagnosis using LFAs requires an additional confirmatory laboratory-based test to correctly establish negative results. Colorimetric-LFAs offer limited quantification ability owing to the limited color change with respect to variation of the target analyte’s concentration.^13^

The COVID-19 pandemic highlights the need for improved LFAs for precise and rapid clinical diagnoses, mass screenings and epidemiological studies.^14–15^ RT-PCR^16–17^ and direct antigen tests^18–19^ have been the mainstay for diagnosis of COVID-19, and serological assays are important for determination of infection stage and vaccine efficacy, and epidemiological studies.^3, 20–21^ These diagnostic platforms are available only in qualified microbiology laboratories and remain expert-dependent, labor-intensive, and time-intensive, limitations that have precluded conduction of the millions of tests per day needed during epidemiological surges.^22–23^ Therefore, a critical need exists for diagnostic and screening tools that are not only as accurate as laboratory-based assays, but also rapid, easy-to-use, inexpensive, readily available (e.g., home-based and POC), and scalable for rapid population-level screening.

Efforts to improve bioanalytical performance of LFAs have included using fluorescent molecules or quantum dots as reporter elements.^24–25^ Although fluorescent reporters improve quantification, their relatively weak signal intensity limits their sensitivity and point-of-care diagnostic utility, and their low light absorption compared to conventional colloidal gold nanoparticles (AuNPs)^26^ precludes the direct visual detection available in conventional LFAs, and moreover requires use of external LFA readers or powerful excitation light sources. These limit the utility of fluorescent LFAs in mass screening and resource-limited settings.^10^

We envision a “bimodal” LFA in which an initial screening can be performed with a visual test, and subsequent quantitative testing can be performed when needed on the same LFA strip using a fluorescence reader. To achieve this, we employed an ultrabright fluorescent nanoconstruct that we have recently introduced,^27^ called plasmonic-fluor, as a bimodal colorimetric and fluorescent reporter in LFAs (Figure 1A). These nanoconstructs harness plasmon-enhanced fluorescence^28–32^ to achieve nearly 7000-fold brighter fluorescence signal compared to conventional molecular fluorophores. We modified plasmonic-fluors with detection antibodies and applied them to enable rapid and ultrasensitive colorimetric and fluorescent detection of analytes, using human IL-6 (LOD: 93 fg ml^−1^), SARS-CoV-2 S1 (subunit of the spike protein) antibodies (LOD: 185 pg ml^−1^), and SARS-CoV-2 antigen (nucleocapsid) protein (LOD: 212 pg ml^−1^). We validated the clinical efficacy of the plasmonic-fluors-based LFAs (p-LFAs) for COVID-19 by testing plasma samples for SARS-CoV-2 S1 antibodies and nasopharyngeal swab samples for nucleocapsid protein SARS-CoV-2 variants-of-concern, respectively, and achieved high analytical specificity (100%) and sensitivity (~97%). Significantly, this technology can also be employed as an alternative to laboratory-based test for the diagnosis of clinically relevant pathogenic infections and possible future pandemics.

## 2. Results And Discussion

### 2.1 Using plasmonic-fluors in LFAs increases sensitivity over AuNPs by 10000-fold

Plasmonic enhancement was first applied to overcome three fundamental limitations of the 30–40 nm AuNPs used as conventional colorimetric labels in LFAs. AuNPs have low capture rate (<5%), low signal-to-background ratio, and thus relatively low sensitivity.^33–34^ Even with use of 100 nm AuNPs, shown recently to improve LFA sensitivity,^35^ these problems persist. Because of these three limitations, color changes in AuNP-based LFAs are limited to qualitative analysis or simply a binary output, indicating the presence or absence of the target analyte.

To assess whether plasmonic-fluors could overcome these limitations, we compared their performance to AuNPs on a nitrocellulose membrane. We set out to determine the minimum number of AuNPs and plasmonic-fluors required to produce a detectable visible or fluorescence signal. When serially diluted AuNPs (Figure 1B) and plasmonic-fluors (Figure 1C) of known particle concentration were drop-casted onto nitrocellulose membrane, accumulations of approximately ~10^6^ nanoparticles were needed to produce a discernable visible signal (Figure 1D and S1). However, only ~10^2^ plasmonic-fluors were required to produce a detectable fluorescence signal (Figure 1E and S2).

Plasmonic-fluors exhibited colorimetric signal nearly identical to that of AuNPs (Figure S3). The colorimetric signal enabled qualitative visual detection (by naked eye), obviating the need for specialized read-out equipment at a relatively high concentration of the target analyte, while the fluorescence signal enabled ultrasensitive detection and quantification of low abundance analytes. Thus, plasmonic-fluor function as a bimodal nanolabel (colorimetric+fluorescent) and offers ultrasensitive detection in a biological detection assay representative of LFAs.

Next, to compare the performance of plasmonic-fluors and AuNPs in LFA format, we employed the well-characterized biotin-streptavidin conjugate pairing, known to exhibit extremely high binding affinity.^36^ Both AuNPs and plasmonic-fluors were functionalized with streptavidin and biotinylated bovine serum albumin (BSA) was used as a capture-ligand. LFA strips were then subjected to different known concentrations of streptavidin-conjugated AuNPs and plasmonic-fluors for 20 min (Figure S4). Nanolabels flows along the nitrocellulose membrane by capillary force and gets captured by the captureligand, leading to the accumulation of nanoparticles at the test spot. Accumulation of sufficient number of nanolabels converts the color at the test site to red, indicating a positive result and the presence of the target analyte. The average greyscale intensity of the colorimetric signal at the test site with AuNPs and the fluorescence signal with plasmonic-fluors monotonically increased with the concentration of the nanolabels (Figure 1G and 1H). Significantly, for both AuNPs and PFs, approximately ~10^7^ nanoparticles are needed to produce a discernable visible signal, however, only ~10^3^ PFs are enough to produce a detectable fluorescence signal. The four-order magnitude lower concentration threshold for a detectable signal with plasmonic-fluors compared to AuNPs in the LFA format is consistent with the drop-casting approach discussed above. These results manifest the fundamental basis that plasmonic-fluors can serve as ultrabright nanolabels for ultrasensitive detection of target analytes in an LFA.

### 2.2 The bioanalytical performance of plasmonic LFAs can be further improved by tuning LFA parameters

Next, we optimized the bioanalytical performance of LFA by tuning concentration of capture ligand and nanolabels. We employed biotin-streptavidin as a model system. Both AuNPs and plasmonic-fluors were biotin functionalized, streptavidin and biotinylated BSA were utilized as target analyte and capture ligand, respectively (Figure 2A and S5). It was observed that as the concentration of capture-ligand (*i.e.*, biotinylated BSA) increased, both mean greyscale intensity and fluorescence intensity of the test spot corresponding to AuNPs (Figure 2B and S6) and plasmonic-fluors (Figure 2C and S7), respectively, increased. These results suggest that higher concentrations of capture-ligand results in better signal intensity. Further, as the number of nanolabels increased, both mean greyscale intensity and fluorescence intensity of the test spot corresponding to AuNPs (Figure 2D and S8) and plasmonic-fluors (Figure 2E and S9), respectively, increased, implying better signal intensity with higher number of nanolabels. However, in both cases, the background signal (signal from the LFA strip outside the capture spot) also increased with the number of nanolabels. Therefore, the optimum number of nanolabels for both AuNPs-based LFA and p-LFA was determined by subtracting the background signal from the test spot signal. As expected, the optimum number of plasmonic-fluors (1.2 × 10^6^) was four orders magnitude lower than the AuNPs (1.78 × 10^10^).

Next, we compared the bioanalytical parameters (limit-of-detection (LOD), limit-of-quantitation (LOQ) and dynamic range) of biotin-streptavidin AuNPs-based LFA and p-LFA. It is worth noting that colorimetric signal, obtained from the 8-bit ImageJ processed images of LFA strips, from both AuNPs (Figures 2F) and plasmonic-fluors (Figures 2G) exhibit similar LOD, suggesting no loss in visual detection capabilities in p-LFAs (Figure S10). The LOD (defined as mean + 3σ of the blank) of AuNPs-based LFA was calculated to be ~ 5 ng ml^−1^ (Figure 2I). In contrast, the fluorescence signal from p-LFA (Figures 2H) enabled the detection down to 11 pg ml^−1^ (Figure 2J, four-parameter logistic fit), representing a 927-fold improvement in the LOD. The LOQ (defined as mean + 10σ of the blank) of p-LFA is ~570-fold better than the LOQ of AuNPs-based LFA. Further, the fluorescent component of plasmonic-fluor augmented the dynamic range of the assay by three orders of magnitude. Therefore, owing to the ultrabright fluorescence signal of the plasmonic-fluors, the p-LFAs enable ultrasensitive detection of target analyte over a much broader range of analyte concentration.

### 2.3 Superior performance of plasmonic-fluors compared to AuNPs LFAs for the detection of human IL-6

Cytokines are small (5–26 kDa) proteins, involved in cell signaling and immuno-modulation and are critical indicators of health and disease.^37^ Several diseases including cancer, sepsis, HIV, chronic inflammation and auto-immune diseases are known to be associated with dysregulation of immune system, leading to disruption of the subtle balance between pro-inflammatory and anti-inflammatory cytokines.^38–39^ The pro-inflammatory cytokines include IL-1 (interleukin-1), IL-6, IL-12, TNFα (tumor necrosis factor α) and IFNγ (interferon γ), while the anti-inflammatory cytokines include TGFβ (transforming growth factor β), IL-10 and IL-4. Rapid monitoring of the immune status by analyzing serum cytokines and early diagnosis of these diseases is essential for prompt clinical intervention and for inhibiting disease progression. Though few LFAs for IL-6 detection have been introduced recently,^40–41^ none provide sensitivity and quantitation comparable to gold-standard ELISA. Therefore, we employed IL-6 as a model target analyte to investigate the applicability of our p-LFA.

**Figure 3:**
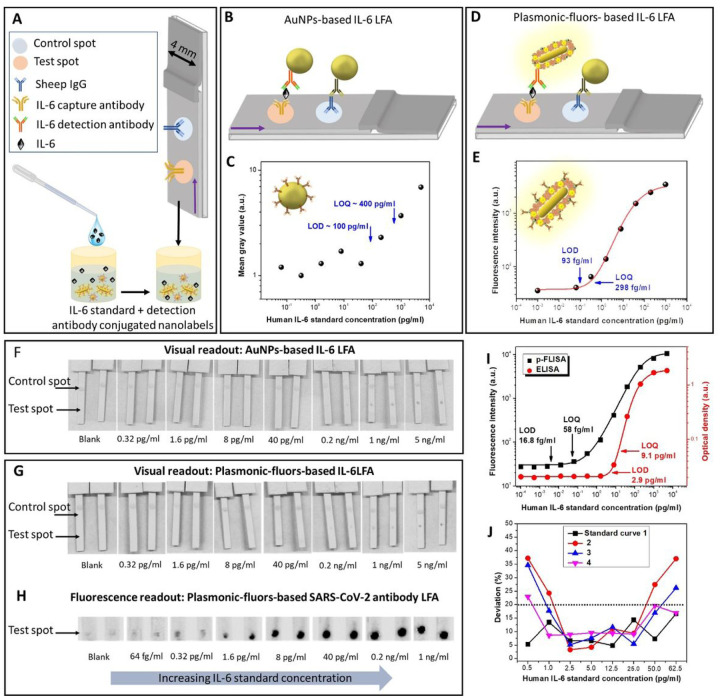
**(A)** Schematic illustration of the IL-6 LFA strips comprising of IL-6 capture antibodies as test spot and sheep IgG as control spot. Schematic description of **(B)** AuNP-based IL-6 LFA and **(D)** IL-6 p-LFA. **(C)** Dose-dependent mean grey values, corresponding to different IL-6 concentrations, acquired from AuNPs-based LFA and **(E)** Dose-dependent fluorescence intensities of IL-6 p-LFA. 8-bit ImageJ processed images of **(F)** AuNP-based IL-6 LFA and **(G)** IL-6 p-LFA depicting the visual readout mode. (**H)** Fluorescence image of the IL-6 p-LFA strips depicting the fluorescence readout mode. **(I)** Dose-dependent optical densities and fluorescence intensities, corresponding to different IL-6 concentrations in standard ELISA (red circle) and plasmonic fluor-linked immunosorbent assay (p-FLISA in black square), respectively, implemented on a microtiter plate, performed in 4 h. **(J)** Plot showing the deviation in the concentration of IL-6 deduced using four different standard curves obtained over a span of six months from the actual concentrations.

Human IL-6 capture antibodies and sheep anti-immunoglobulin G (IgG) antibodies were immobilized on a nitrocellulose membrane to form test and control spots, respectively (Figure 3A and S11). The LOD of AuNPs-based LFA (Figure 3B) was calculated to be ~ 100 pg ml^−1^ (Figure 3C). In contrast, the fluorescence signal intensity obtained from p-LFA (Figure 3D) enabled the detection down to 93 fg ml^−1^ (Figure 3E, four-parameter logistic fit), which represents a 1075-fold improvement in the LOD compared with conventional AuNPs-based LFAs and Significantly higher than the recently reported LFAs^40–41^. The LOQ of p-LFA (300 fg ml^−1^) is ~1300-fold better than the LOQ of AuNPs-based LFA (400 pg ml^−1^). Further, the plasmonic-fluor improved the dynamic range of the LFA by nearly three-order magnitude. The colorimetric signal from both AuNPs and p-LFA exhibited similar LODs, suggesting no loss in visual detection capabilities in p-LFAs (Figure 3F, 3G and S12). Additionally, the fluorescence signal from the plasmonic-fluors enabled ultrasensitive detection and quantitative analysis over a much broader range of analyte concentration (Figure 3E and 3H).

We also compared the sensitivity and LOD of p-LFA with standard ELISA and plasmonic-fluor linked immunosorbent assay (p-FLISA) (Figure S13) implemented on a microtiter plate. The LOD of p-LFA is nearly 30-fold lower compared to conventional sandwich ELISA (2.9 pg ml^−1^) and only 5-fold inferior to that of p-FLISA (16.8 fg ml^−1^) (Figure 3I). However, the sample-to-answer time for p-LFAs was 20 min whereas ELISA and p-FLISA require 4 h.

Next, to determine the quantitative detection ability of p-LFA, multiple IL-6 standard curves were acquired over a span of six months (Figure S14). Using these standard curves, IL-6 concentration ranging from 1 pg ml^−1^ to 50 pg ml^−1^ were accurately quantified with less than 20% deviation (Figure 3J and S15). The ability to accurately quantify the analyte concentration, which has not been reported with LFA platforms, ascertains that p-LFAs enable quantitative detection of target analyte in standard-free manner. Thus, p-LFAs overcome the long-standing limitation of LFAs – limited sensitivity, low accuracy and smaller analytical range compared to laboratory tests, and limited quantitation ability.

### 2.4 p-LFA allows for SARS-CoV-2 antibody detection with the accuracy and sensitivity of ELISA

To assess the potential for clinical translation of our p-LFA, we next optimized it for detection of SARS-CoV-2 antibodies. A pressing need persists for sensitive, rapid and POC serological assays for SARS-CoV-2, both for epidemiological studies and for vaccine efficacy against SARS-CoV-2 studies.^3, 20^ Several LFAs^3–4, 42^ and other assay platforms^43^ exist that employ SARS-CoV-2 spike protein as recognition element for detection of SARS-CoV-2 antibodies. Using p-LFA, our goal was to extend the sensitivity and limit of detection beyond the range possible with current assays, and into the range of ELISA.

**Figure 4:**
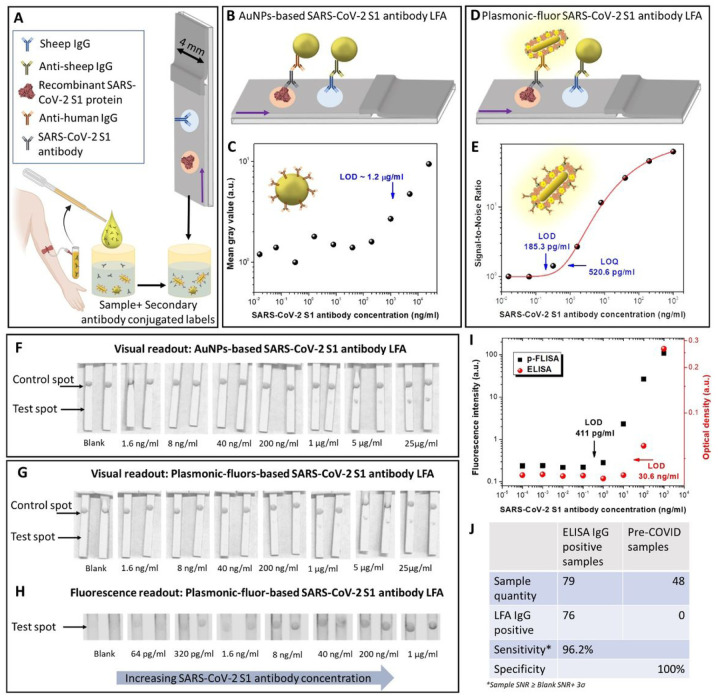
**(A)** Schematic illustration of the SARS-CoV-2 S1 antibody LFA strips comprising of recombinant SARS-CoV-2 S1 protein as capture element at the test spot and sheep IgG at the control spot. Schematic description of **(B)** AuNP-based SARS-CoV-2 S1 antibody LFA and **(D)** p-LFA. **(C)** Dose-dependent mean grey values, corresponding to different SARS-CoV-2 S1 antibody concentrations, acquired from AuNPs-based LFA and **(E)** Dose-dependent signal-to-noise ratio of SARS-CoV-2 S1 antibody p-LFA performed in 20 min. 8-bit ImageJ processed images of **(F)** AuNP-based SARS-CoV-2 S1 antibody LFA and **(G)** SARS-CoV-2 S1 antibody p-LFA depicting the visual readout mode. **(H)** Fluorescence image of the SARS-CoV-2 S1 antibody p-LFA strips depicting the fluorescence readout mode. **(I)** Dose-dependent optical densities and fluorescence intensities, corresponding to different SARS-CoV-2 S1 antibody concentrations, obtained by standard ELISA (red circle) and p-FLISA (black square) implemented on a microtiter plate, performed in 4 h. **(J)** Table depicting the analytical sensitivity and specificity of the SARS-CoV-2 S1 antibody p-LFA.

Recombinant SARS-CoV-2 S1 subunit of spike protein was immobilized at the test spot and sheep IgG was used for control spot (Figure 4A and S16). We first determined the bioanalytical parameters of AuNPs-based LFA (Figure 4B) and to p-LFA (Figure 4D) for detection of SARS-CoV-2 S1 antibody. Using the colorimetric signal obtained from LFA strips, the LOD of AuNPs-based LFA was determined to be ~ 1.2 μg ml^−1^ (Figure 4C). In contrast, p-LFA exhibited an LOD of 185 pg ml^−1^ (Figure 4E, four-parameter logistic fit), which represents a nearly 6500-fold improvement. Further, as expected, the mean greyscale intensities obtained from both AuNPs and p-LFA exhibited similar sensitivity, suggesting no compromise in the visual detection capabilities (Figure 4F, 4G and S17). However, the fluorescence signal from plasmonic-fluors enabled ultrasensitive detection and quantitative analysis over a much broader (3 to 4 orders of magnitude higher) range of analyte concentration (Figure 4E and 4H). p-LFA displayed 165-fold improvement in LOD as compared to conventional sandwich ELISA and comparable LOD to p-FLISA (Figure 4I).

To assess the translational potential of p-LFAs, we tested 79 plasma samples obtained from COVID-19 positive individuals and 48 archived de-identified serum/plasma samples which were collected pre-COVID-19 (March-October 2019) under HRPO 201102546^44^ for the presence of SARS-CoV-2 S1 antibodies. All 127 plasma samples were diluted 500-fold and tested using p-LFA. Out of 79 IgG positive samples (tested positive by ELISA), 76 were tested positive (sample SNR ≥ blank SNR + 3σ of blank) with LFA, indicating 96.2% sensitivity. All pre-COVID-19 samples tested negative with LFA for SARS-CoV-2 S1 IgGs, indicating 100% specificity (Figure 4J). Thus, the p-LFAs for SARS-CoV-2 antibodies detection offers POC applicability with accuracy comparable to gold standard ELISA and with potential applicability to vaccine efficacy and epidemiological studies.

### 2.5 p-LFA allows for sensitive SARS-CoV-2 antigen detection

Finally, we evaluated the potential of p-LFAs to fill the critical need for a POC test that can provide information about whether a patient is currently infectious. State of the art diagnosis of common respiratory virus infections does not achieve this. The challenge is that in serological testing of virus-specific immunoglobulins, the antibody responses to viral antigens are usually detected in the late stage of infection (7–14 days after virus exposure), therefore serological antibody tests cannot achieve accurate screening of asymptomatic populations or early stages of infection.^45^ Further, RT-PCR, the current gold standard in diagnosing COVID-19, has proven highly successful in identifying individuals who have contracted the SARS-CoV-2 virus, however, they fail to distinguish between infectious patients and noninfectious individual, and can yield false positive results for months after a patient has recovered from the disease.^46–47^

Since antigens are expressed only when the virus is actively replicating, the antigen-based tests have better correlation with infectiousness than RNA detection by RT-PCR. Current antigen detection tests for diagnosing COIVD-19 are scalable and convenient but are limited by their low and wide ranging accuracy.^48–51^ LFAs for detection of SARS-CoV-2 antigens can be the most important tool owing to their ease of use, lower-cost and better correlation with infectivity. Currently, several LFA-based antigen^6–7, 52^ assays have been reported and are widely used but none offers the optimal sensitivity,^53^ thus, a negative result with such platforms in a symptomatic patient requires a confirmatory RT-PCR test. Therefore, there is an urgent need for a more sensitive POC antigen assay that would be just as reliable and accurate as the RT-PCR method.

p-LFA provided the accuracy and sensitivity needed for this in samples from patients who simultaneously had PCR tests performed. Our test focused on the detection of SARS-CoV-2 nucleocapsid protein (N protein). Test and the control spots on the LFA strips were prepared by immobilizing N protein capture antibodies and sheep IgG, respectively (Figure 5A). The fluorescence signals obtained from p-LFAs increased monotonically with an increase in the concentration of N protein standard (Figure 5B). The LOD and LOQ were calculated to be 212 pg ml^−1^ and 1.02 ng ml^−1^, respectively (Figure 5C, four-parameter logistic fit). The p-LFA displayed 37-fold improvement in LOD as compared to conventional sandwich ELISA and comparable LOD to p-FLISA (Figure S19).

Next, to demonstrate the clinical translational potential of p-LFAs for detection of N protein, we tested 35 PCR-positive samples, comprised of 16 delta variant samples, and 19 PCR-negative NP swab samples. The negative NP swab samples comprised a mix of healthy samples, and samples tested positive for seasonal coronaviruses and other respiratory viruses. All the 19 PCR-negative samples tested negative (SNR < mean + 3σ) via p-LFAs, suggesting 100% analytical specificity to COVID-19 N protein and no cross-reactivity with other viruses and different seasonal coronaviruses. Of the 35 PCR positive samples, 34 tested positive with p-LFAs (SNR > mean + 3σ), indicating 97.1% analytical sensitivity (Figure 5D).

Further, to demonstrate the advantage of p-LFAs over an existing commercial FDA EUA approved rapid, point-of-care antigen testing platform, we compared the analytical sensitivity of p-LFAs with BD Veritor^™^ assay. It indicated samples with concentrations below 50 ng/ml as “Presumptive Negative.” This indicates nearly 235-fold better analytical sensitivity of p-LFA compared to the commercial antigen test. p-LFA outperformed the FDA-approved BD Veritor^™^ antigen kit when analyzing PCR-positive COVID-19 patient samples. Overall, it correctly identified 8 out of 19 positive NP swab samples, corresponding to analytical sensitivity of only 42.1% (Figure 5D). While 13/14 patient samples in the early stage of illness (<10 days since symptoms onset) tested positive by p-LFA (93% sensitivity), only 7 tested positive by BD Veritor^™^ (50% sensitivity) (Table S1). The diagnostic sensitivity of p-LFA for samples with low viral load (cycle threshold (CT) values ≥ 25) is 91.7% (11 out of 12) and for samples with high viral load (CT values < 25) is 100% (23 out of 23). This is Significantly higher than the previously reported rapid antigen/POC SARS-CoV-2 tests’ diagnostic sensitivity (~80% for samples with CT values < 25 and 20–40% for samples with CT values ≥ 25).^7, 53–55^ These results substantiate that p-LFAs enable ultrasensitive, accurate, rapid, inexpensive, and point-of-care diagnosis of COVID-19 antigen and antibodies and thus can be a potential tool for rapidly and precise diagnosis of symptomatic and asymptomatic infections.

## 3. Conclusions

In summary, plasmonic-fluors were demonstrated as a bimodal (colorimetric+fluorescent) reporter element for overcoming long-standing limitations of LFAs: limited sensitivity, low accuracy and small dynamic range compared to laboratory tests, and limited quantitation ability. Plasmonic-fluors produced a discernable fluorescence signal at densities 10000-fold lower than those needed in conventional colorimetric AuNPs. p-LFAs for various analytes (IL-6, SARS-CoV-2 S1 antibodies, and SARS-CoV-2 antigen) exhibited ~1000-fold improvement in bioanalytical parameters (LOD, LOQ and dynamic range). p-LFAs offered standard-free quantitative detection and the sensitivity of gold standard ELISA, but with a much lower sample-to-answer time (20 min versus 4–6 hours). p-LFAs for detection of COVID-19 antibodies and antigens present in plasma and nasopharyngeal swab samples, respectively, achieved >95% sensitivity and 100% specificity, demonstrating its clinical applicability. We believe p-LFAs are highly attractive for point-of-care settings with better accuracy than conventional colorimetric LFAs, and with faster sample-to-answer time and lower cost than molecular tests. The platform technology demonstrated here can be readily adapted for the detection of other infectious pathogens and disease biomarkers, and can be employed as an alternative to laboratory-based test for the diagnosis of clinically relevant pathogenic infections and possible future pandemics.

## Methods

### Synthesis of plasmonic-fluors

Plasmonic-fluors consists of plasmonically active core, gold nanorod synthesized by seed-mediated method,^56^ a polymer spacer layer, fluorophores and universal biorecognition element (biotin). Plasmonic-fluors were synthesized following the similar procedure described in our previous study.^27^ Detailed stepwise procedure is discussed in the supplementary information.

### Synthesis of gold nanoparticles (AuNPs)

Citrate-stabilized AuNPs were synthesized using seed-mediate synthesis method and using citrate as reducing agent. Au seeds (~15 nm) were synthesized as described previously by Frens et al.^57^ Briefly, 20 ml 0.25 mM of HAuCl_4_ (Sigma Aldrich, 520918) was brought to boil under vigorous stirring, 800 rpm. Immediately after the solution started boiling, 0.2 ml of 3% (w/v) sodium citrate (Sigma Aldrich, 1613859) aqueous solution was added and maintained under boiling condition until the solution color changed to wine red, indicating the formation of Au seeds. Next ~100 nm AuNPs were synthesized using hydroquinone (Sigma Aldrich, H9003) as reducing agent for reduction of ionic gold.

### Materials characterization

TEM images were obtained using a JEOL JEM-2100F field emission instrument. The extinction spectra of plasmonic nanostructures were obtained using a Shimadzu UV-1800 spectrophotometer. Fluorescence mappings were recorded using LI-COR Odyssey CLx imaging system. Digital camera (Sony cybershot DSC HX300) and imaging software, ImageJ were employed to characterize mean gray intensities. SpectraMax iD3 (Molecular Devices) plate reader was used to measure the optical density in ELISA.

### Functionalization of nanolabels

To functionalize nanolabels with streptavidin (Sigma Aldrich, SA101), 1 μl 10 mg ml^−1^ of streptavidin (or BSA-Biotin or detection antibody) was added to 1 ml OD1 of nanolabels and incubated for 1 h on a shaker at room temperature. To stabilize the particles, 1 ul 10 mg ml^−1^ of BSA (Sigma Aldrich, A7030) was added to the solution and further incubated for 20 min. Unbound protein was removed by washing the solution four times with pH 10 nanopure water (1 μl NaOH in 10 ml of water). Finally, nanolabels were redispersed in 1% BSA in 1X PBS solution for use in the LFAs. To functionalize nanolabels with antibodies (IL-6 and N protein detection antibody and anti-human IgG), similar process was employed.

### Lateral flow immunoassay assembly and preparation procedures

Nitrocellulose test membrane and absorbent pads with adhesive backing material (GE healthcare, FF120HP) were employed for fabricating the LFA strips. The test membrane and absorbent pad was cut into 4 mm wide strips using a paper trimmer. For preparing the LFA strip, biorecognition element (e.g., capture antibody) solution was pipetted onto the test membrane and dried at room temperature for 30 min. Subsequently, the test membrane was blocked using 3% BSA in 1X PBS solution. Next, strips were washed with PBST (1X PBS and 0.5% Tween20 (Sigma Aldrich, P9416), followed by drying at room temperature in a vacuum desiccator for 1 h. After drying, absorbent pads (GE healthcare, CF5) were assembled onto the polystyrene adhesive backing next to nitrocellulose test membrane. To ensure efficient transfer of the solution from the test membrane to the absorbent pad, we ensured an overlap of 1–2 mm between both strips. Experiments were performed by dipping LFAs into 96-well plates filled with 100 μl of sample/standard solutions for 20 min. The visual signals of LFAs were obtained by a digital camera. The images were converted to 8-bit gray scale image using ImageJ. Mean gray values of the test spot were calculated by averaging the test spot grayscale intensities obtained from ImageJ. The fluorescence signals were obtained by averaging test dot fluorescence intensities obtained using LI-COR Odyssey CLx fluorescence scanner using the following scan parameters: laser power~L2; resolution 21 μm; channel 800 nm; height 0 mm.

### Optimization of lateral flow immunoassay parameters

To determine the optimum concentration of biotinylated BSA on the test spot, different LFA strips with varying concentrations of biotinylated BSA (100 μg ml^−1^ to 5 mg ml^−1^) were prepared. LFAs were then subjected to the same concentration of streptavidin (1000 ng ml^−1^ for AuNP-LFA and 1 ng ml^−1^ for p-LFA) and biotinylated nanolabels. To determine the optimal concentration of the nanolabels, LFA strips with the same concentration of the capture element and biotinylated BSA (5 mg ml^−1^) were prepared. These LFA strips were then subjected to the same concentration of streptavidin (1000 ng ml^−1^ for AuNPs and 1 ng ml^−1^ for plasmonic-fluors) but different numbers of biotin-functionalized nanolabels (4.45×10^6^ to 3.56×10^10^ for AuNPs and 1.2×10^4^ to 6×10^6^ for plasmonic-fluors). The optimum number of nanolabels for both AuNPs-based LFA and p-LFA was determined by subtracting the background signal from the test spot signal.

### Biotin-streptavidin lateral flow immunoassay

Test spots were formed by pipetting 0.5 μl of 5 mg ml^−1^ biotinylated-BSA onto the nitrocellulose membrane. The LFA strip was assembled as described above. LFA strips were dipped into microtiter wells filled with 100 μl of different concentrations of streptavidin solutions (0.1 pg ml^−1^ to 1000 μg ml^−1^) for 20 min.

### Human IL-6 immunoassays

Human IL-6 DuoSet ELISA kit (R&D systems, DY206) was utilized in the study. For AuNP-based IL-6 LFA, AuNPs were conjugated with IL-6 detection antibody for the test spot and with anti-sheep IgG (R&D systems, BAF016) for the control spot. For p-LFA, plasmonic-fluors were conjugated with IL-6 detection antibody for the test spot and AuNPs were conjugated with anti-sheep IgG for the control spots, respectively. To prepare LFA strips for IL-6 immunoassay, 0.5 μl of 2 mg ml^−1^ IL-6 capture antibody and 0.5 μl of 2 mg ml^−1^ sheep IgG (R&D systems, 5–001-A) was pipetted onto the nitrocellulose membrane at different spots to create test and control spot, respectively. Subsequently similar steps, mentioned above, were followed for LFA preparation and assembly. For AuNP-based IL-6 LFA, 1 μl of IL-6 detection antibody-conjugated AuNPs and 1 μl of anti-sheep IgG conjugated-AuNPs for test and control spot, respectively, were mixed with 98 μl of different concentrations of human IL-6 standard solutions (100 fg ml^−1^ to 5 ng ml^−1^) in 96-well plates to allow the binding of the analyte with the detection antibody-conjugated nanolabels. LFA strips were then exposed to the sample/standard solution for 20 min. For IL-6 p-LFA, 1 μl of IL-6 detection antibody-conjugated plasmonic-fluors and 1 μl of anti-sheep IgG conjugated AuNPs were mixed with 98 μl of human IL-6 standard solutions (1 fg ml^−1^ to 1 ng ml^−1^) in 96-well plates. The visual signals and the fluorescence signals were obtained according to the procedure described above.

Human IL-6 ELISA was carried out according to the procedure described in DuoSet ELISA kit manual and is discussed in detail in supplementary information. Plasmonic fluor-linked immunosorbent assay (p-FLISA) was carried out by adopting a similar approach, expect that the HRP-labeled streptavidin (provided in the ELISA kit) was replaced by streptavidin-functionalized plasmonic-fluor. Instead of streptavidin-HRP, 100 μl of streptavidin-plasmonic-fluors (OD 1) was incubated for 30 min, and then the plate was washed three times with PBST. The fluorescence signal was obtained by averaging the fluorescence intensities from the microtiter wells obtained using LI-COR Odyssey CLx with the following scan parameters: laser power~L2; resolution 169 μm; channel 800 nm; height 4 mm.

### Lateral flow immunoassay quantitation study

Four IL-6 standard curves were generated over a span of 6 months and samples with varying IL-6 concentrations (0.5 pg ml^−1^ to 62.5 pg ml^−1^) were tested in a standard-free manner. Their experimental concentrations were determined using each standard curve, and deviation from actual concentrations were calculated.

### SARS-CoV-2 S1 antibody immunoassays

We pipetted 0.5 μl of 2 mg ml^−1^ recombinant SARS-CoV-2 S1 protein (R&D systems, 10522-CV) and 0.5 μl of 2 mg ml^−1^ sheep IgG onto the nitrocellulose membrane as test and control spot, respectively. Subsequently, we followed the same steps described above to prepare the LFA strips. For detecting SARS-CoV-2 S1 antibodies, AuNP-LFA and p-LFA, AuNPs and plasmonic-fluors were conjugated with biotinylated anti-human IgG for test spots, respectively. In both cases, AuNPs were conjugated with anti-sheep IgG for control spot. For AuNP-based SARS-CoV-2 S1 antibody LFA, 1 μl of anti-human IgG conjugated-AuNPs and 1 μl of anti-sheep IgG conjugated-AuNPs were mixed with different concentrations of standard solutions (16 pg ml^−1^ to 25 μg ml^−1^) in 96-well plates, prior to exposure to LFA strip for 20 min.

For plasmonic-fluor-based SARS-CoV-2 S1 antibody LFA, 1 μl of anti-human IgG conjugated-plasmonic-fluors and 1 μl of anti-sheep IgG conjugated-AuNPs were mixed with different concentrations of standard solutions (16 pg ml^−1^ to 1 μg ml^−1^) in 96-well plates, prior to exposure to LFA strip for 20 min. Plasma samples were diluted 500-fold in reagent diluent (1X PBS containing 3% BSA, 0.2 μm filtered) before use. The visual signals and the fluorescence signals were obtained by employing the same procedure mentioned above.

SARS-CoV-2 S1 antibody ELISA was carried out according to the following procedure. Microtiter wells were coated with 100 μl of 5 μg ml^−1^ (in 1X PBS) recombinant SARS-CoV-2 S1 protein via overnight incubation at room temperature. For blocking, 300 μl of reagent diluent was added to the wells for a minimum of 1 h. Next, 100 μl of serially-diluted standard samples were incubated for 2 h, followed by incubation of 100 μl of 100 ng ml^−1^ biotinylated anti-human IgG for 2 h. Next, 100 μl of 500 ng ml^−1^ streptavidin-labelled HRP (Thermo Fisher scientific, N100) was incubated for 20 min, followed by the addition of 100 μl of substrate solution for 20 min. The reaction was stopped by addition of 50 μl of 2N H_2_SO_4_ (R&D Systems, DY994) and immediately the optical density at 450 nm was measured using a microplate reader. p-FLISA was carried out by adopting a similar procedure, expect that the HRP-labelled streptavidin was replaced by streptavidin functionalized-plasmonic-fluor. Instead of HRP, 100 μl of plasmonic-fluors (OD 1) were incubated for 30 min, and then the plate was washed three times with PBST. The fluorescence signal was obtained by averaging the fluorescence intensities from the microtiter wells obtained using LI-COR Odyssey CLx.

### SARS-CoV-2 antigen (nucleocapsid protein) immunoassays

We pipetted 0.5 μl of 2 mg ml^−1^ nucleocapsid protein capture antibodies (SinoBiologicals, 40143-MM08) and 0.5 μl of 2 mg ml^−1^ sheep IgG onto the nitrocellulose membrane as test and control spots, respectively. For N protein p-LFA, plasmonic-fluors were conjugated with biotinylated N protein detection antibody for the test spots. AuNPs conjugated with anti-sheep IgG were employed for control spot. Subsequently, similar steps mentioned above were followed to prepare and assemble the LFA strips. For plasmonic-fluor-based N protein LFA, 1 μl of detection antibodies conjugated-plasmonic-flours and 1 μl of anti-sheep IgG conjugated-AuNPs were incubated with different concentrations of standard solution (12 pg ml^−1^ and 1 μg ml^−1^; SinoBiologicals, 40588-V08B) in 96-well plates prior to exposure to LFA strips for 20 min. p-LFAs were employed for the detection of N protein present in patient nasal swab samples. The nasal swab samples were in universal transport media and were used without any dilution or processing. The visual signals and the fluorescence signals were obtained employing the similar process described above.

N protein ELISA was carried out by first coating the microtiter wells with 100 μl of 100 ng ml^−1^ N protein capture antibodies (in 1X PBS) via overnight incubation at room temperature. For blocking, 300 μl of reagent diluent was added to the wells for a minimum of 1 h. Next, 100 μl of serially-diluted standard samples were incubated for 2 h, followed by incubation of 100 μl of 200 ng ml^−1^ biotinylated N protein detection antibody for 2 h. Next, 100 μl of 500 ng ml^−1^ streptavidin-labelled HRP (Thermo Fisher scientific, N100) was incubated for 20 min, followed by the addition of 100 μl of substrate solution for 20 min. The reaction was stopped by addition of 50 μl of 2N H_2_SO_4_ (R&D Systems, DY994) and immediately the optical density at 450 nm was measured using a microplate reader. p-FLISA was carried out by adopting a similar procedure, expect that the HRP-labelled streptavidin was replaced by streptavidin-functionalized plasmonic-fluor. Instead of HRP, 100 μl of plasmonic-fluors (OD 1) were incubated for 30 min, and then the plate was washed three times with PBST. The fluorescence signal was obtained by averaging the fluorescence intensities from the microtiter wells obtained using LI-COR Odyssey CLx.

### Analysis of patient samples

The clinical samples used in the study were acquired from the repository of saliva and nasopharyngeal samples from individuals confirmed/suspected with COVID-19 disease, located at Washington University School of Medicine in St Louis, and from the Barnes Jewish Clinical Microbiology Laboratory. Control NP swab samples from asymptomatic healthy volunteers were obtained with prior written consent. For evaluation of cross reactivity with seasonal coronaviruses, samples were obtained from adults at Barnes-Jewish Hospital who were tested positive with either of the four seasonal coronaviruses or respiratory diseases via clinically warranted NP samples tests. Washington University School of Medicine Human Research Protection Office (HRPO) approved the study. All clinical data pre-existed at the time of data collection. A prior waiver of consent was obtained for the clinical information and data on COVID-19 PCR results.

### Commercial antigen test

BD Veritor kit, Veritor System – For Rapid Detection of SARS-CoV-2, was used to analyze the presence of N protein in the patient samples. BD Veritor System was used in conjunction with the BD Veritor Plus Analyzer. Nasal swabs were eluted in Universal Transport Media (UTM) and Aimes (ESwab) transport medium. Internal validation and the assay precision was conducted and deemed acceptable for testing on clinical samples by the Barnes Jewish Clinical Microbiology Laboratory.

## Supplementary Material

Supplement 1

## Figures and Tables

**Figure 1:** (**A)** Schematic illustration of plasmonic-fluor, employed as a bimodal nanolabel (colorimetric+fluorescent) in LFAs, comprising of gold nanorod as plasmonic core, polymer as spacer layer, molecular fluorophores and universal recognition element, biotin. Transmission electron microscopy image of **(B)** AuNPs and **(C)** plasmonic-fluors. **(D)** Mean grey value obtained from nitrocellulose membrane drop-casted with different concentrations of AuNPs. Inset shows the 8-bit ImageJ processed image of the nitrocellulose membrane. **(E)** Fluorescence intensity obtained from nitrocellulose membrane drop-casted with different concentrations of plasmonic-fluors. Inset shows the fluorescence image of nitrocellulose membrane. **(F)** Visible-NIR extinction spectra of AuNPs and plasmonic-fluors conjugated with streptavidin. **(G)** Mean grey values obtained from nitrocellulose membranes, with biotinylated-BSA used as capture-ligand at test sites, after exposure to different concentrations of streptavidin-conjugated AuNPs. Inset shows the schematic illustration of streptavidin-conjugated AuNPs. **(H)** Fluorescence intensities obtained from nitrocellulose membranes, with biotinylated-BSA as recognition elements at test sites, after exposure to different concentrations of streptavidin-conjugated plasmonic-fluors. Inset shows the schematic illustration of streptavidin-conjugated plasmonic-fluors. Purple arrows indicate the direction of flow. Error bars represent standard deviations from four different samples (n=4).

**Figure 2:** **(A)** Schematic illustration of the streptavidin-biotin LFA. **(B)** Mean grey value and **(C)** fluorescence intensity of the test spot, obtained from nitrocellulose membranes with different concentrations of biotinylated BSA, after exposure to identical concentration of target analyte (streptavidin) and nanolabels to determine the optimum capture element concentration. **(D)** Mean grey value and **(E)** fluorescence intensity of the test spot, obtained from nitrocellulose membranes with identical concentration of biotinylated BSA, after exposure to identical concentration of target analyte but different concentrations of nanolabels to determine the optimum number of nanolabels. 8-bit ImageJ processed images of **(F)** AuNPs-based streptavidin-biotin LFA and **(G)** streptavidin-biotin p-LFA depicting the visual readout mode. **(H)** Fluorescence image of the streptavidin-biotin p-LFA strips depicting the fluorescence readout mode. **(I)** Dose-dependent mean grey values, corresponding to different streptavidin concentrations, acquired from AuNPs-based LFA and **(J)** Dose-dependent fluorescence intensities of streptavidin-biotin p-LFA.

**Figure 5:** **(A)** Schematic illustration of the nucleocapsid protein p-LFA strips comprising of N protein capture antibody as test spot and sheep IgG as control spot. **(B)** Fluorescence image of the N protein p-LFA strips. **(C)** Dose-dependent signal-to-noise ratio, corresponding to different N protein concentrations, acquired from N protein p-LFA, performed in 20 min. **(D)** Relative signal-to-noise ratio of N protein p-LFA for 19 PCR-negative, 19 PCR-positive samples, and 16 PCR positive NP swab samples for SARS-CoV-2 variant B.1.617.2 (delta). PCR negative NP swab samples are comprised of a mix of samples from healthy individuals, and samples from individuals who tested positive for seasonal coronaviruses and other respiratory viruses.
