# Supplementary material for: Plasmon-enhanced Quantitative Lateral Flow Assay for Femtomolar Detection of SARS-CoV-2 Antibodies and Antigens
Source: Res Sq. 2022 Feb 21:rs.3.rs-1258688. Preprint. [Version 1] doi: 10.21203/rs.3.rs-1258688/v1 (PMC8863156; doi:10.21203/rs.3.rs-1258688/v1)
Supplement: Supplement 1 [file 92b7c47e4ffc804e72a1922f.docx]

**Plasmon-enhanced Quantitative Lateral Flow Assay for Femtomolar Detection of SARS-CoV-2 Antibodies and Antigens**

Rohit Gupta^1^, Prashant Gupta^1^, Zheyu Wang^1^, Anushree Seth^1^, Jeremiah J. Morrissey^2,3^, Ige George^4^, Sumanth Gandra^4^, Gregory A. Storch^5^, Bijal A. Parikh^6^, Guy M. Genin^1,7^, Srikanth Singamaneni^1,3*^

^1^Department of Mechanical Engineering and Materials Science, Institute of Materials Science and Engineering, Washington University in St. Louis, St Louis, MO, 63130, USA.
^2^Department of Anesthesiology, Division of Clinical and Translational Research, Washington University in St. Louis, St. Louis, MO, 63110, USA
^3^Siteman Cancer Center, Washington University School of Medicine, St. Louis, MO, 63110, USA
^4^Department of Internal Medicine, Division of Infectious Diseases, Washington University School of Medicine, St. Louis, MO, 63110, USA
^5^Department of Pediatrics, Washington University School of Medicine, St. Louis, MO, 63110, USA
^6^Department of Pathology and Immunology, Washington University School of Medicine, St. Louis, MO, 63110, USA
^7^NSF Science and Technology Center for Engineering MechanoBiology, Washington University in St. Louis, St. Louis, MO, 63130, USA

*To whom correspondence should be addressed: [singamaneni@wustl.edu](mailto:singamaneni@wustl.edu)

Table of contents

[Synthesis of plasmonically active core, gold nanorods: 3](#_Toc91607131)

[Fluorescence enhancement of 800CW–streptavidin using AuNR–plasmonic-fluor–800CW: 5](#_Toc91607132)

[Human IL-6 enzyme-linked immunosorbent immunoassay: 5](#_Toc91607133)

[Biotinylation of anti-human IgG: 6](#_Toc91607134)

[Biotinylation of N protein detection antibody: 6](#_Toc91607135)

[Antibody conjugation on plasmonic-fluor: 6](#_Toc91607136)

[Figures 7](#_Toc91607137)

[Figure S1 7](#_Toc91607138)

[Figure S2 8](#_Toc91607139)

[Figure S3 9](#_Toc91607140)

[Figure S4 10](#_Toc91607141)

[Figure S5 11](#_Toc91607142)

[Figure S6 12](#_Toc91607143)

[Figure S7 13](#_Toc91607144)

[Figure S8 14](#_Toc91607145)

[Figure S9 15](#_Toc91607146)

[Figure S10 16](#_Toc91607147)

[Figure S11 17](#_Toc91607148)

[Figure S12 18](#_Toc91607149)

[Figure S13 19](#_Toc91607150)

[Figure S14 20](#_Toc91607151)

[Figure S15 21](#_Toc91607152)

[Figure S16 22](#_Toc91607153)

[Figure S17 23](#_Toc91607154)

[Figure S18 24](#_Toc91607155)

[Figure S19 25](#_Toc91607156)

[Figure S20 26](#_Toc91607157)

[Table 1 27](#_Toc91607158)

# Synthesis of plasmonically active core, gold nanorods:

Plasmonic-fluors were synthesized following the similar procedure described in our previous study.^1^ To prepare plasmonic-fluor specific to 800CW dye, first AuNRs with absorbance wavelength 760 nm (localized surface-plasmon resonance wavelength ~760 nm) were synthesized by the seed-mediated method.^2^ If needed, the wavelength of gold nanorods can be easily tuned to couple with different dye molecules to achieve the best enhancement factor. Briefly, to a solution of 9.75 ml 0.1 M hexadecyltrimethylammonium bromide (CTAB) (Sigma Aldrich, H5882), 0.25 ml 10 mM HAuCl_4_ (Sigma Aldrich, 520918) and ice-cold 0.6 ml 10 mM NaBH_4_ solution (Sigma Aldrich, 71321) was added under vigorous stirring at room temperature for 5 minutes or until the solution color changed to brown from yellow indication reduction of gold salt and formation of gold seed. Next to synthesize gold nanorods, 380 ml 0.1 M CTAB, 20 ml 0.01 M HAuCl_4_ aqueous solution, 5.5 ml 0.01 M AgNO_3_ (Sigma Aldrich, 20439 0), 8 ml 1 M HCI (Sigma Aldrich, H9892), 2.2 ml 0.1 M ascorbic acid (Sigma Aldrich, A92902) and 5 µl of seed solution were added sequentially. Each addition is followed by gentle homogenization and after last addition the subsequent solution is left in dark for 24 h. Finally, the solution was centrifuged at 7,000 rpm for 30 mins to remove excess reactants and was then reconstituted in nanopure water for further use.

*Conjugation procedures (800CW-BSA-Biotin)*

Using EDC–NHS chemistry, first BSA was conjugated with biotin. For this, solution of 2.2 ml 5 mg ml^−1^ BSA (Sigma Aldrich, A7030) in 1XPBS and 2 mg NHS–PEG4–biotin (Thermo Scientific, 21329) was incubated at room temperature for 1 h. Conjugated BSA-biotin was purified by a desalting column (Thermo Scientific, 21329, 7,000 MWCO). Next to conjugate 800CW to BSA-biotin, 100 µl 1M of K_2_HPO_4_ buffer (to raise pH of the solution to 9) was added to 1 ml of purified BSA-biotin solution. Next, 25 µl 4 mg ml^−1^ NHS–800CW (LI-COR, 929–70020) was added to the above solution, and then incubated at room temperature for 2 h. Finally, conjugated BSA–biotin–800CW was purified using a Zeba desalting column pre-equilibrated with nanopure water.

*Synthesis of plasmonic-fluor*

AuNR (760 nm) was employed as the nanoantenna to prepare plasmonic fluor–800CW. 1 µl of (3-mercaptopropyl)trimethoxysilane (MPTMS) (Sigma Aldrich, 175617) and 1 ml of AuNR (extinction ~2) were mixed and shaken on rocking bed for 1 h. Next, 2 µl of APTMS (Sigma Aldrich, 281778) and 2 µl of trimethoxy(propyl)silane (TMPS) (Sigma Aldrich, 662275) was added to the MPTMS-modified AuNR to form the polymer spacer layer. Excess monomers were removed from the AuNR–polymer solution by two centrifugations at 6000 rpm for 10 min. After each wash the pellet was redispersed in 1 M CTAB aqueous solution. Polymer coated AuNRs were concentrated into a final volume of 10 µl. Next, to conjugate 800CW-BSA-Biotin complex to polymer modified AuNRs, we followed procedures mentioned in previously reported study.^3^ Briefly, to allow coating of 800CW-BSA-Biotin to AuNRs, the pH of 100 µl 4 mg ml^−1^ BSA–biotin–800CW was lowered by adding 1 µl of 20 mg ml^−1^ citric acid (Alfa Aesar, 36664). To this solution, concentrated AuNR-polymer solution was added, and the resulting solution was sonicated for 20 min in dark. After coating, excess 800CW-BSA-Biotin was removed by centrifugation at 3,000 rpm for 10 min and incubated with 0.4 mg ml^−1^ of 800CW-BSA-Biotin in pH 10 nanopure water (1 µl NaOH in 10 ml of water) for 3 d at 4 °C. Finally, the nanostructures were washed 4 times using ph10 nanopure water by centrifugation at 3,000 rpm for 10 mins. Finally, nanolabels were redispersed in 1% BSA in 1X PBS solution for use in immunoassays.

*Concentration measurements of nanolabels*

The concentration of AuNPs and plasmonic-fluors were determined according to Beer’s law:$A= \varepsilon\cdot c \cdot l.$Here, *A* is the local surface plasma resonance (LSPR) absorbance of nanolabels measured by UV-VIS spectroscopy, 𝑐 is the concentration of nanolabels, 𝑙 is the light path length (cm), 𝜀 is the molar extinction coefficient (M^-1^∙cm^-1^) of nanoparticles described below: 10^(1.0643*log (1.5*3.14159*diameter) +4.0935)^.

Fluorescence enhancement of 800CW–streptavidin using AuNR–plasmonic-fluor–800CW:
The schematic of procedure adopted for the test is depicted in Supplementary figure 20. Specifically, first 100 µl of 50 ng ml^−1^ of BSA–biotin (in 1X PBS) was incubated at room temperature for 15 min. The wells were washed three times with 350 µl of PBST (0.05% Tween 20 in 1X PBS) followed by blocking with 300 µl of reagent diluent (1X PBS containing 3% BSA, 0.2 µm filtered). Next the wells were incubated with 1 µg ml^−1^ of streptavidin–800CW (LI-COR, 926-32230), diluted in reagent diluent, for 10 min. The wells were washed again with PBST and incubated with just PBS (for control) and 76 pM plasmonic-fluor in reagent diluent for 30 min. To remove unbound or excess plasmonic-fluors, the wells were washed again thrice with PBST. Subsequently, the fluorescence signal of well with and without plasmonic-fluors were measured using the LI-COR CLx fluorescence imager with the following scanning parameters: laser power ~L2; resolution ~169 µm; channel 800; height 4 mm.

# Human IL-6 enzyme-linked immunosorbent immunoassay:

Human IL-6 ELISA was carried out according to the procedure mentioned in DuoSet ELISA kit manual. Specifically, wells were first coated with 100 µl 2 µg ml^−1^ (in 1X PBS) of capture antibodies via overnight incubation at room temperature. Next for blocking, 300 µl of reagent diluent (1X PBS containing 3% BSA, 0.2 µm filtered) was incubated in wells for minimum of 1 h. Next, 100 µl of serially diluted standard samples were incubated for 2 h, followed by incubation of 100 µl 50 ng ml^−1^ biotinylated detection antibodies for 2 h. Next, 100 µl of streptavidin labelled horseradish peroxidase (200-fold diluted using reagent diluent) was incubated for 20 min, followed by incubation of 100 µl of substrate solution, 1:1 mixture of color reagent A (H_2_O_2_) and B (tetramethylbenzidine) (R&D Systems, DY999) for 20 min. The reaction was stopped by addition of 50 µl 2N H_2_SO_4_ (R&D Systems, DY994) and immediately after the optical density at 450 nm was measured using a microplate reader.

# Biotinylation of anti-human IgG:

Using EDC–NHS chemistry, antibodies were conjugated with biotin. First NHS-PEG4-biotin solution was prepared by addition of 170 µl of nanopure water in 2 mg of NHS-PEG4-biotin (Thermo Scientific, 21329). Next, mixture of 1 ml 2 mg ml^−1^ of anti-human IgG (Rockland, 609-4617) in 1X PBS and 30 µl NHS-PEG4-biotin solution were incubated at room temperature for 1 h. Biotinylated anti-human IgG was purified by 7,000 MWCO desalting column (Thermo Scientific, 21329).

# Biotinylation of N protein detection antibody:

Using EDC–NHS chemistry, N protein detection antibodies were conjugated with biotin. First NHS-PEG4-biotin solution was prepared by addition of 170 µl of nanopure water in 2 mg of NHS-PEG4-biotin (Thermo Scientific, 21329). Next, mixture of 400 µl of as received N protein detection antibody (SinoBiologicals, 40143-R004) in 1X PBS and 5 µl NHS-PEG4-biotin solution were incubated at room temperature for 1 h. Biotinylated N protein detection antibody was purified by 7,000 MWCO desalting column.

# Antibody conjugation on plasmonic-fluor:

Streptavidin-conjugated plasmonic-fluor (40 µl, extinction 32) was added to 50 µl of 4.5 µg/ml biotinylated anti-human IgG and biotinylated detection antibody for N protein at room temperature. The solution was incubated for 0.5 h and washed thrice with pH 10 water. For washing conjugated plasmonic-fluor was centrifuged at 6,000 rpm (2800 g) for 10 minutes. Finally, the pellet was resuspended in 1% BSA in 1x PBS and stored in 4 C for further use in immunoassays.

# Figures


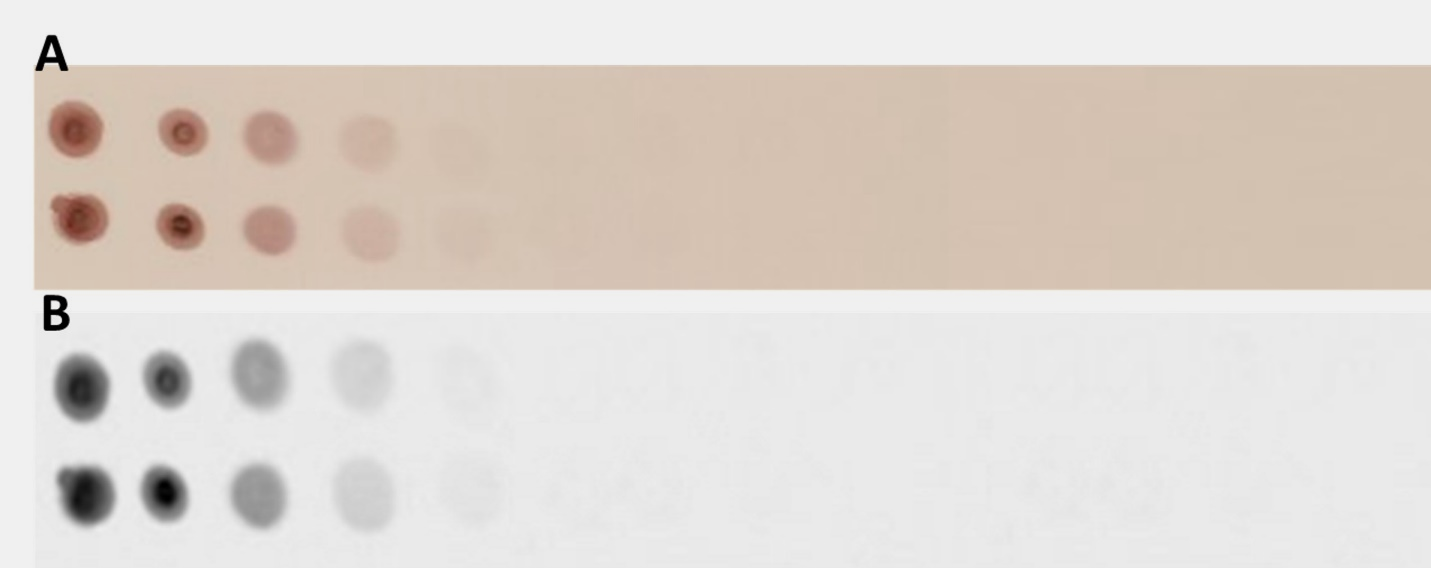


Figure S1**:** (A) Digital photograph of a strip drop-casted with different concentrations of AuNPs. (B) 8-bit ImageJ processed image.


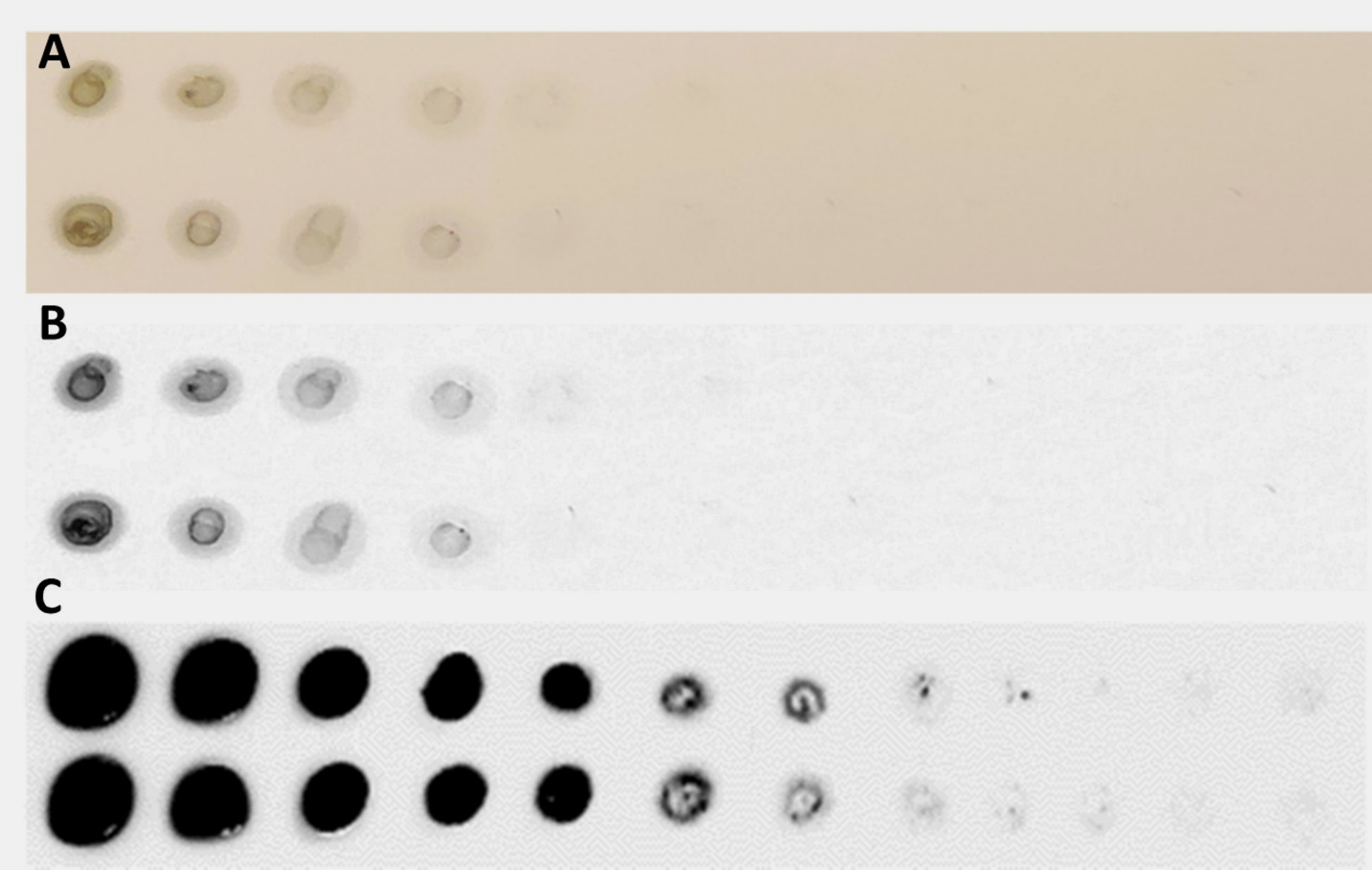


Figure S2**:** (A) Digital photograph of the strip drop-casted with different concentrations of plasmonic-fluors. (B) 8-bit ImageJ processed image. (C) Corresponding fluorescence image of the nitrocellulose membrane.


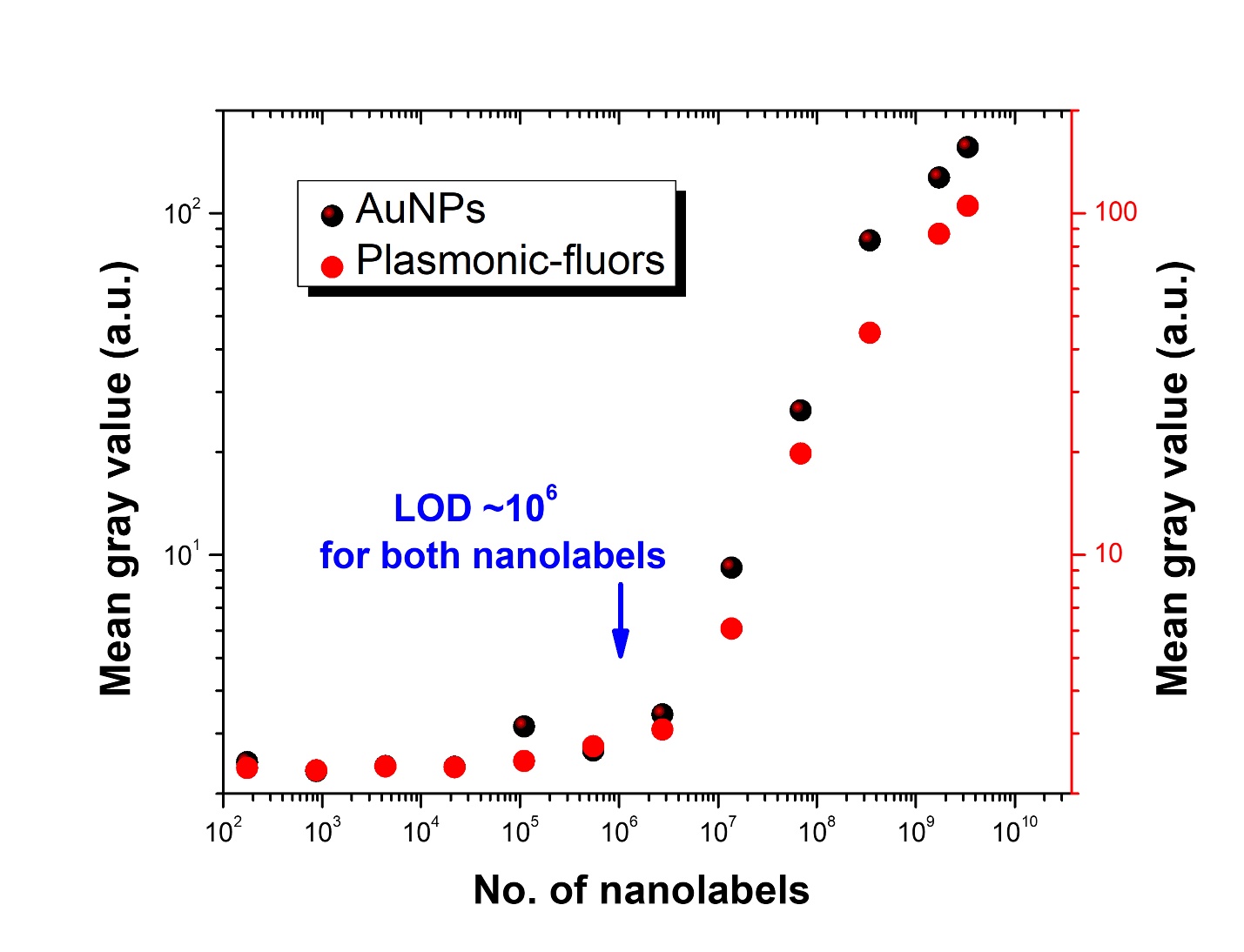


Figure S3**:** (A) Mean gray values obtained from nitrocellulose membrane drop-casted with different concentrations of AuNPs and plasmonic-fluors.


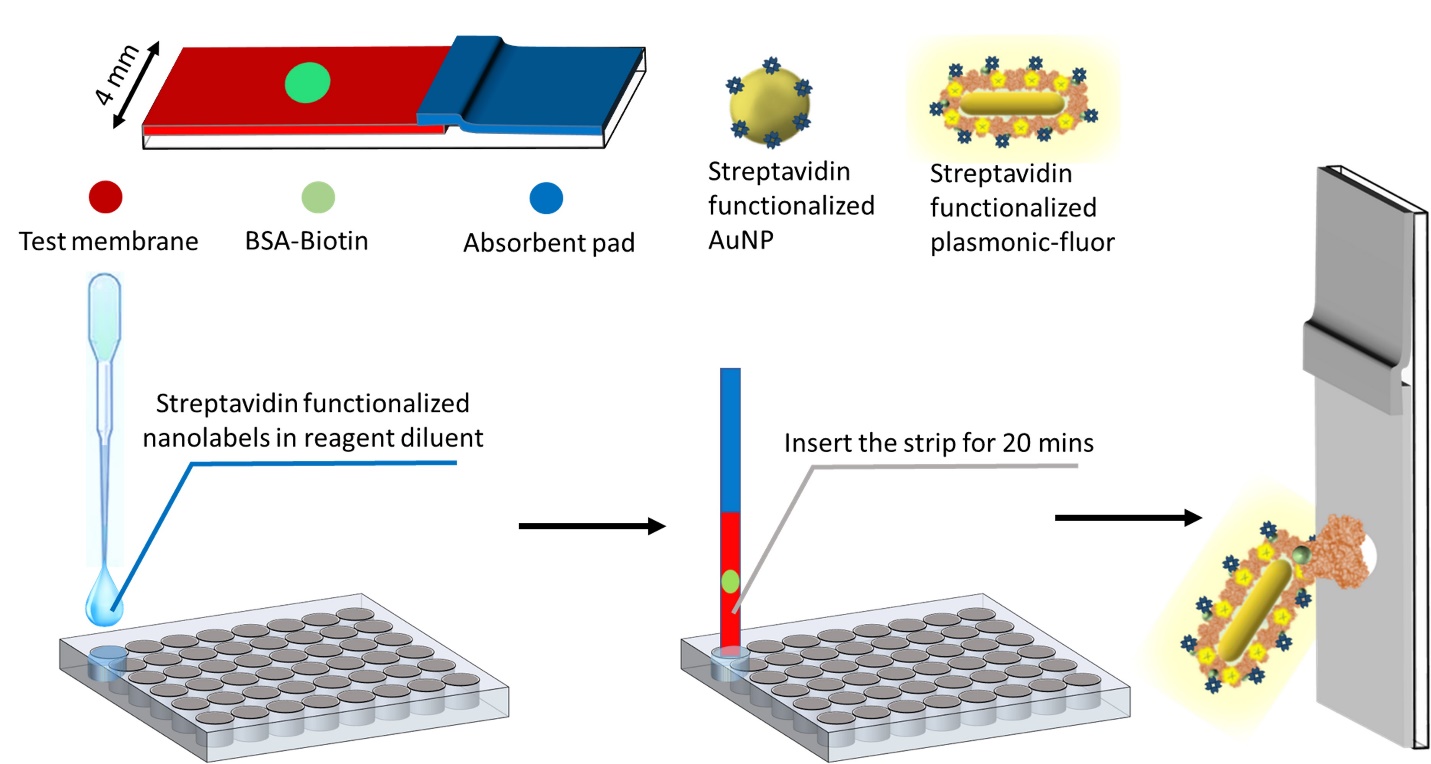


Figure S4: Schematic representation of the experiment conducted to determine minimum concentration of nanolabels needed to produce detectable signal on nitrocellulose membrane in LFA format.


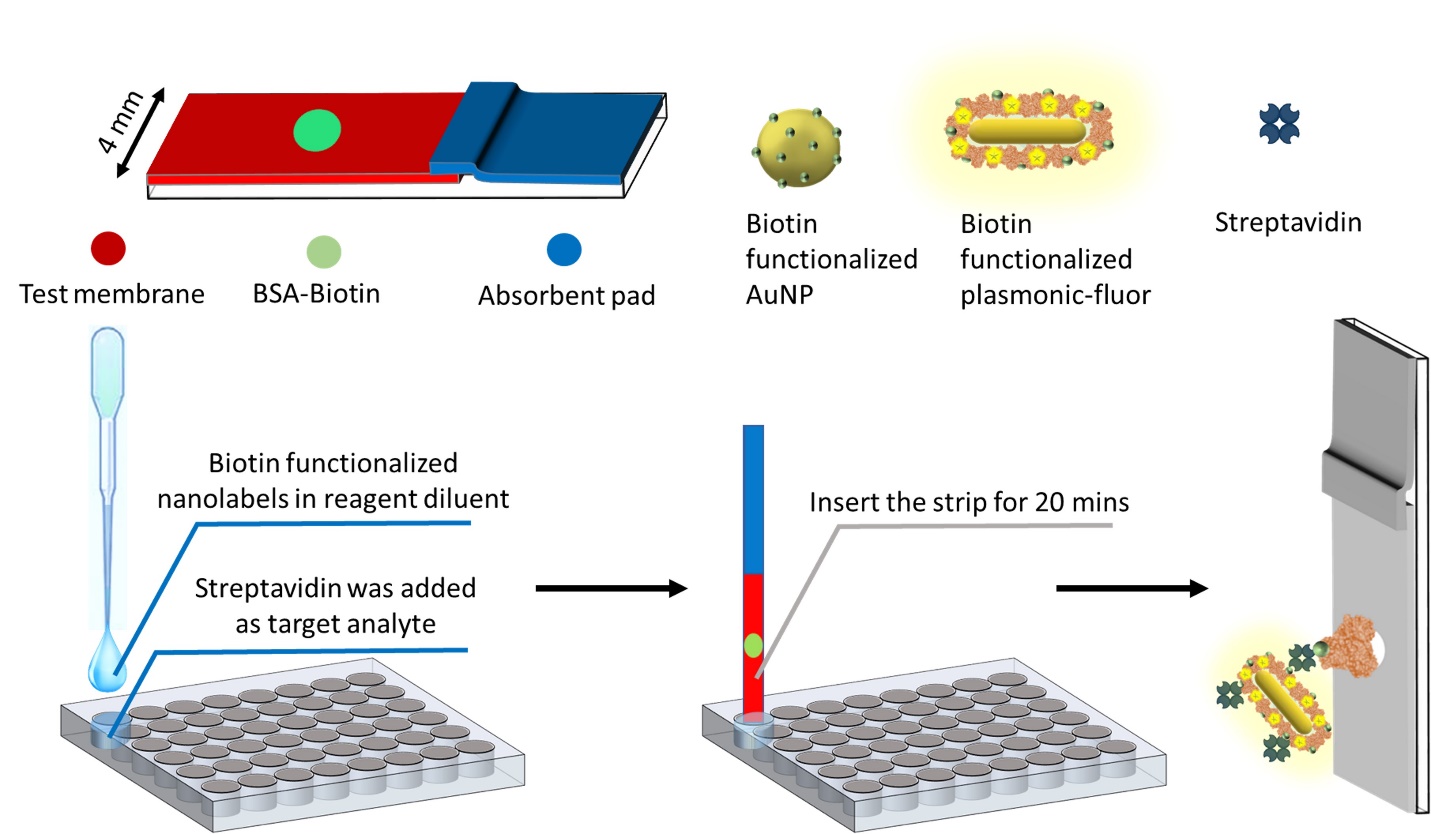


Figure S5**:** Schematic representation of biotin-streptavidin lateral flow assay.


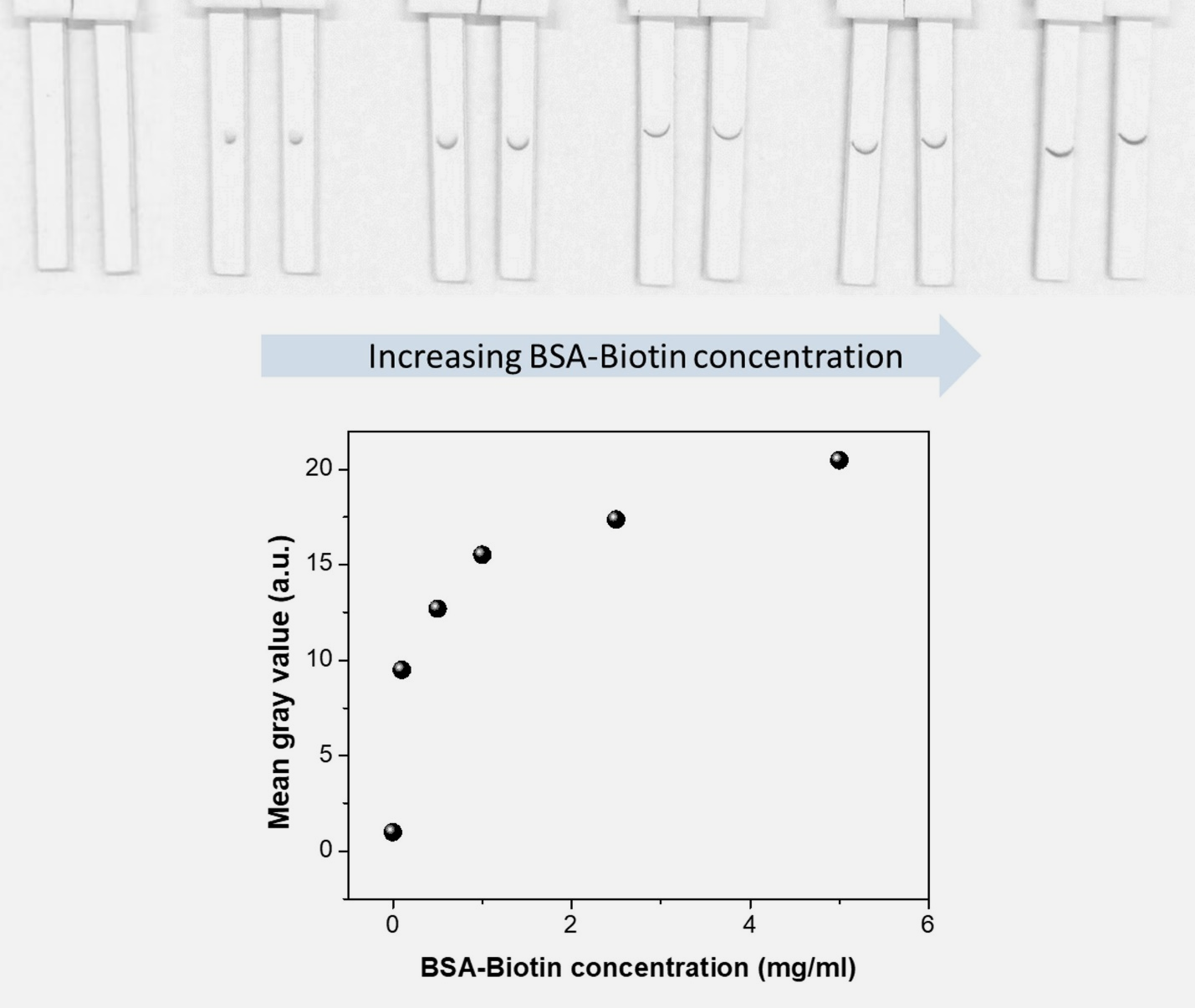


Figure S6**:** (Top) Image of the LFA strips (after Image J processing), with different concentrations of BSA-Biotin as capture spot, after exposure to the same concentration of streptavidin as target analyte and biotin-functionalized AuNPs. (Bottom) Mean gray value as a function of BSA-Biotin concentration.


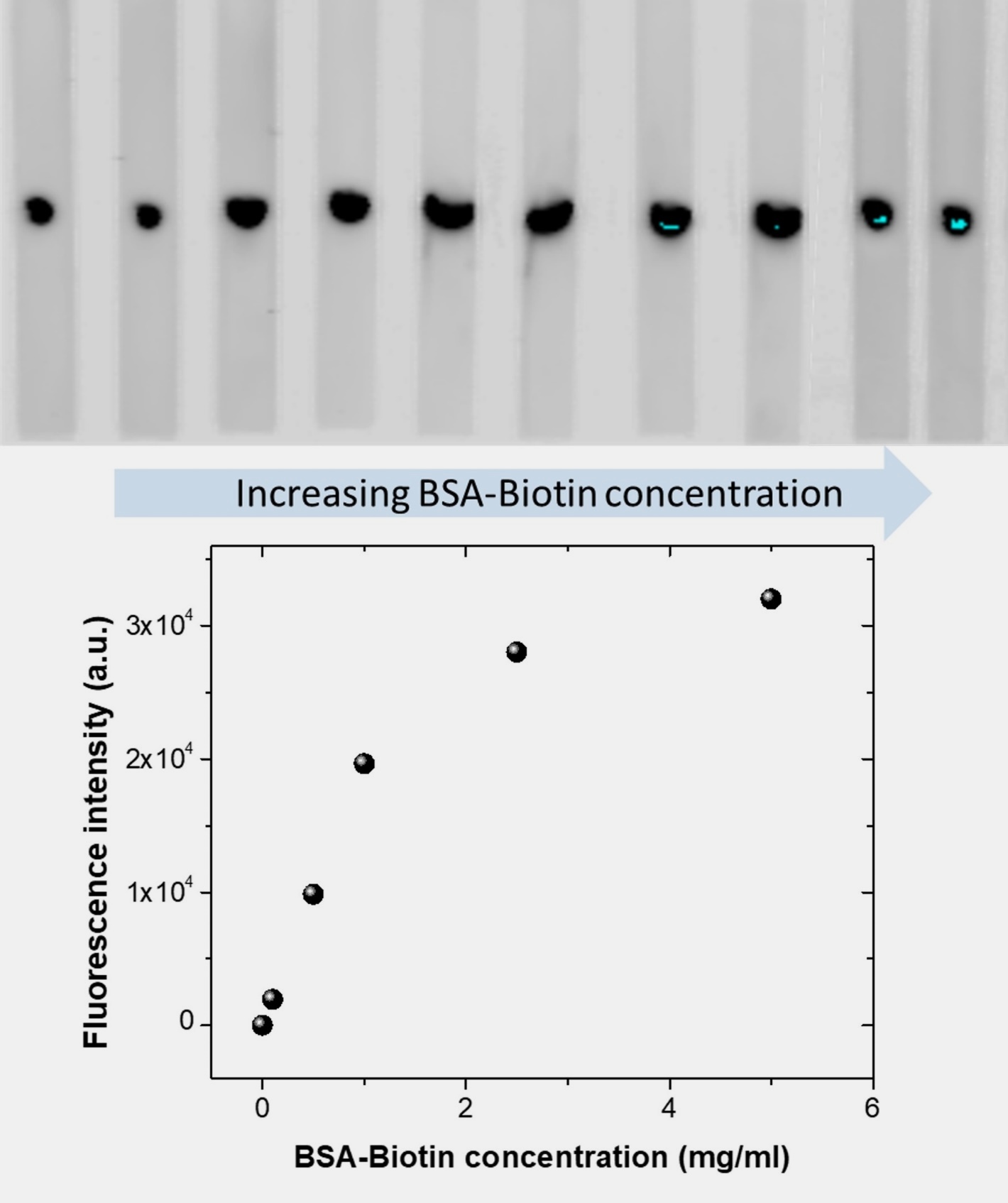


Figure S7**:** (Top) Fluorescence image of the LFA strips, with different concentrations of BSA-Biotin as capture spot, after exposure to the same concentration of streptavidin as target analyte and biotin-functionalized plasmonic-fluors. (Bottom) Fluorescence intensity as a function of BSA-Biotin concentration.


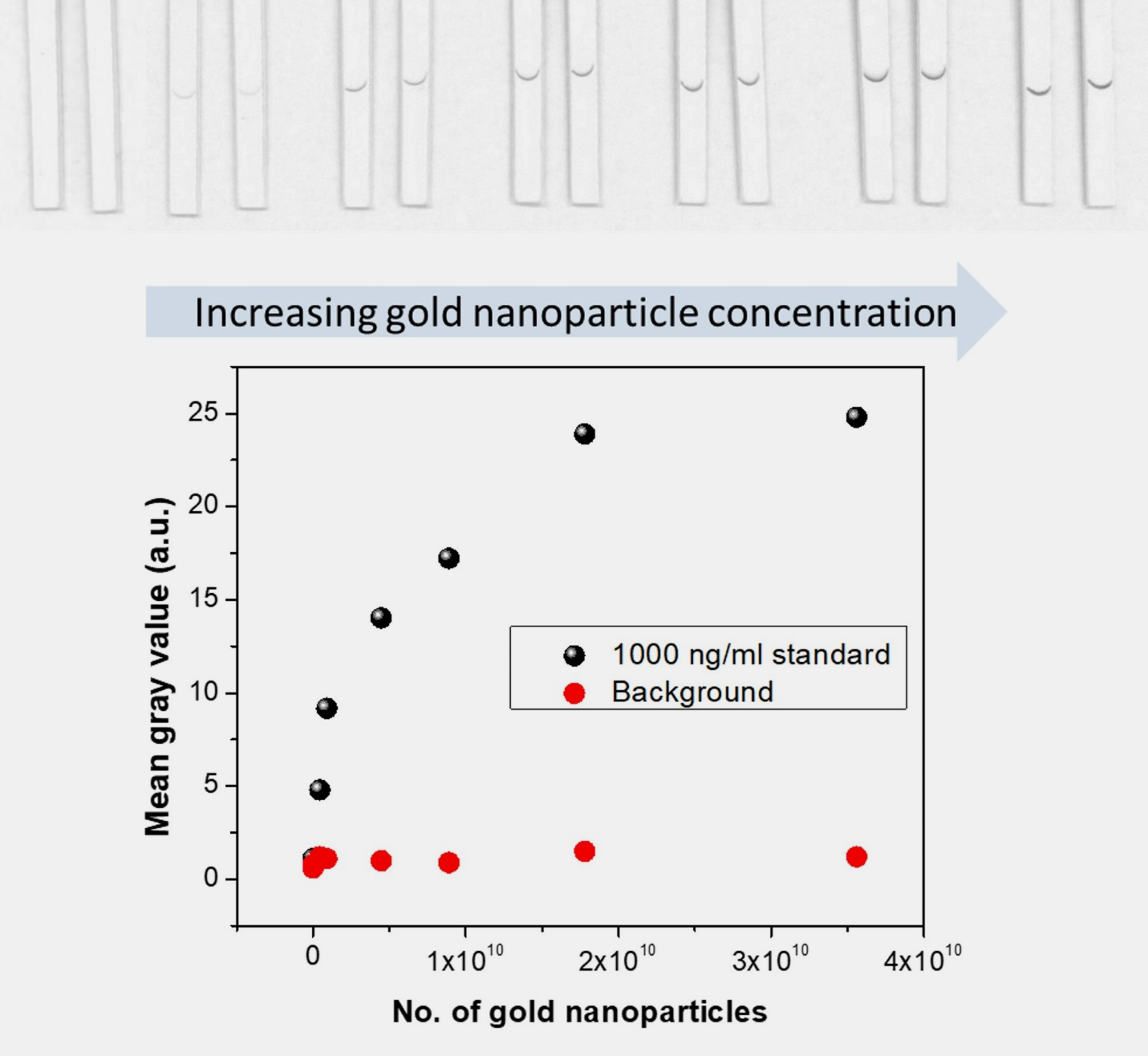


Figure S8**:** (Top) Image of LFA strips (Image J processed) with the same concentration of BSA-Biotin concentration after exposure to different concentrations of biotin functionalized AuNPs, and identical concentrations of streptavidin as target analyte. (Bottom) Mean gray values as a function of AuNP concentrations.


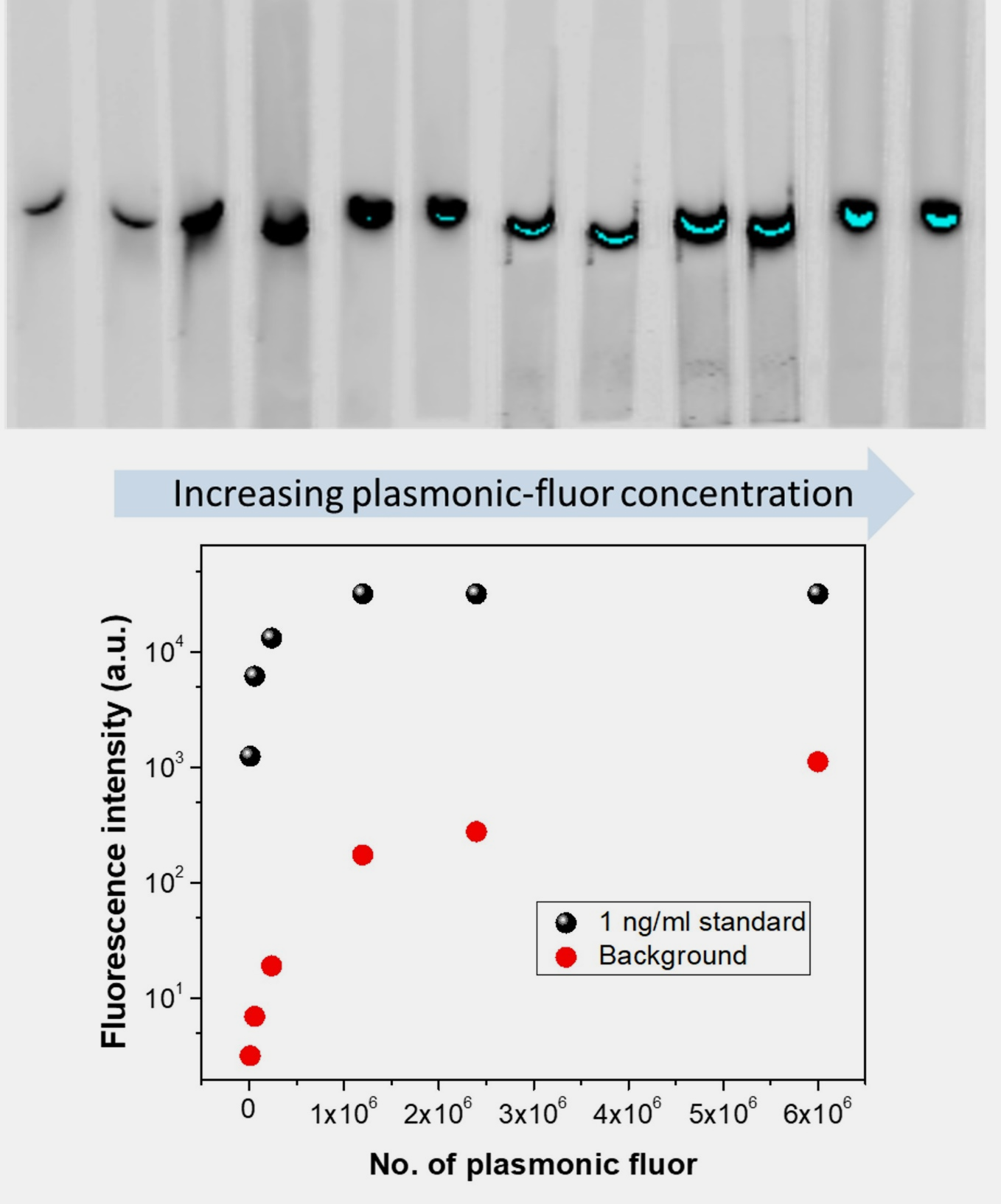


Figure S9**:** (Top) Fluorescence image of LFAs with the same concentration of BSA-Biotin concentration after exposure to different concentrations of biotin functionalized plasmonic-fluors, and identical concentrations of streptavidin as target analyte. (Bottom) Fluorescence intensity as a function of plasmonic-fluors concentration.


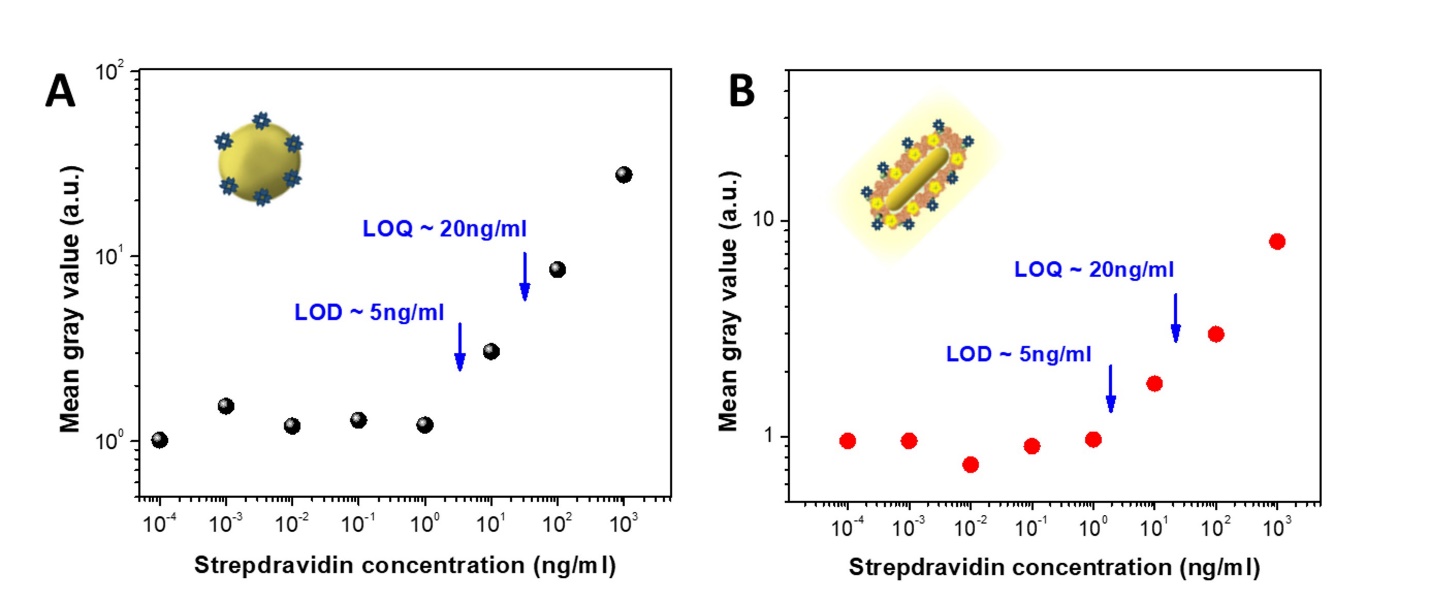


Figure S10**:** Dose-dependent mean gray values obtained from nitrocellulose membrane corresponding to different concentrations of streptavidin acquired from (A) AuNP-based and (B) plasmonic-fluor-based biotin-streptavidin LFA.


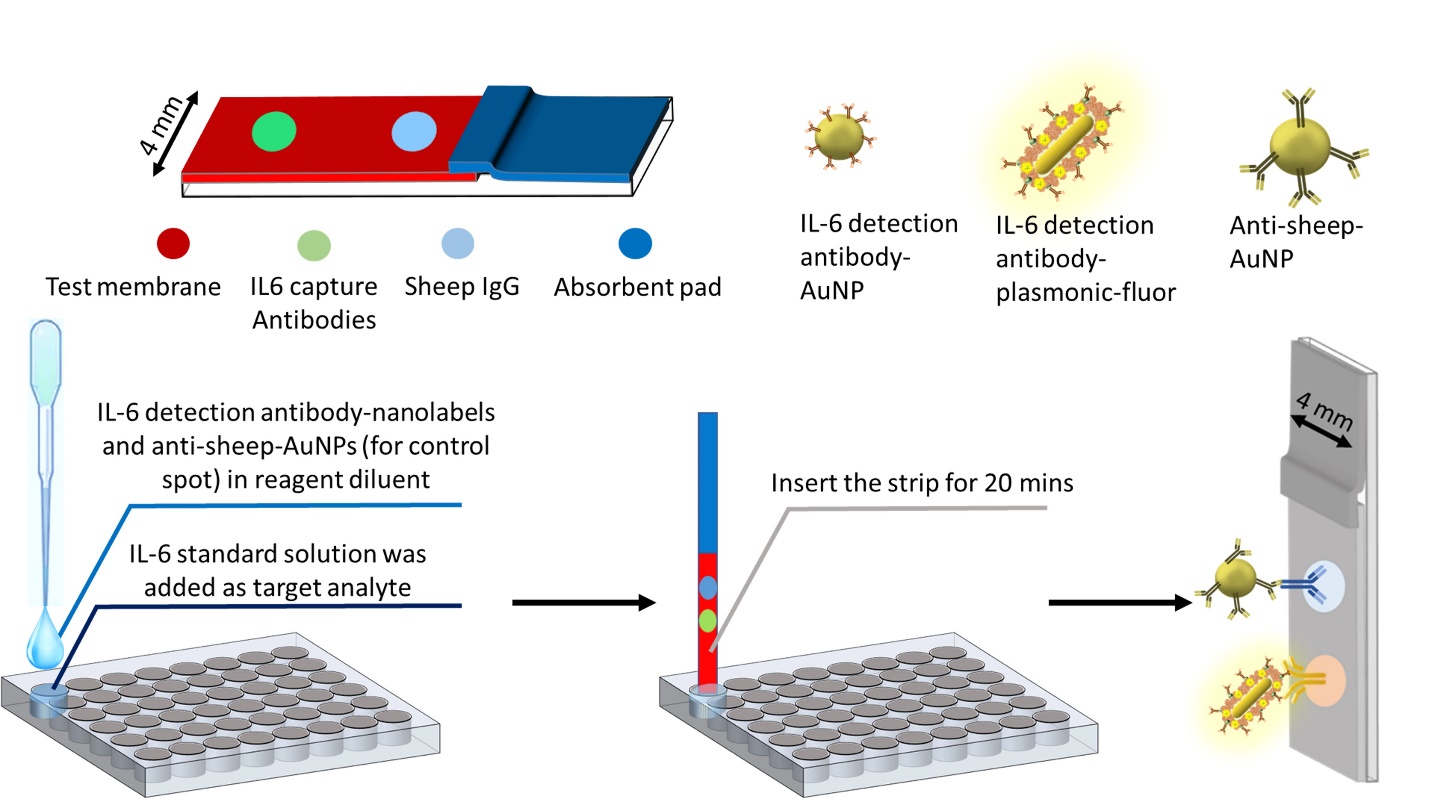


Figure S11**:** Schematic representation of IL-6 lateral flow assay.


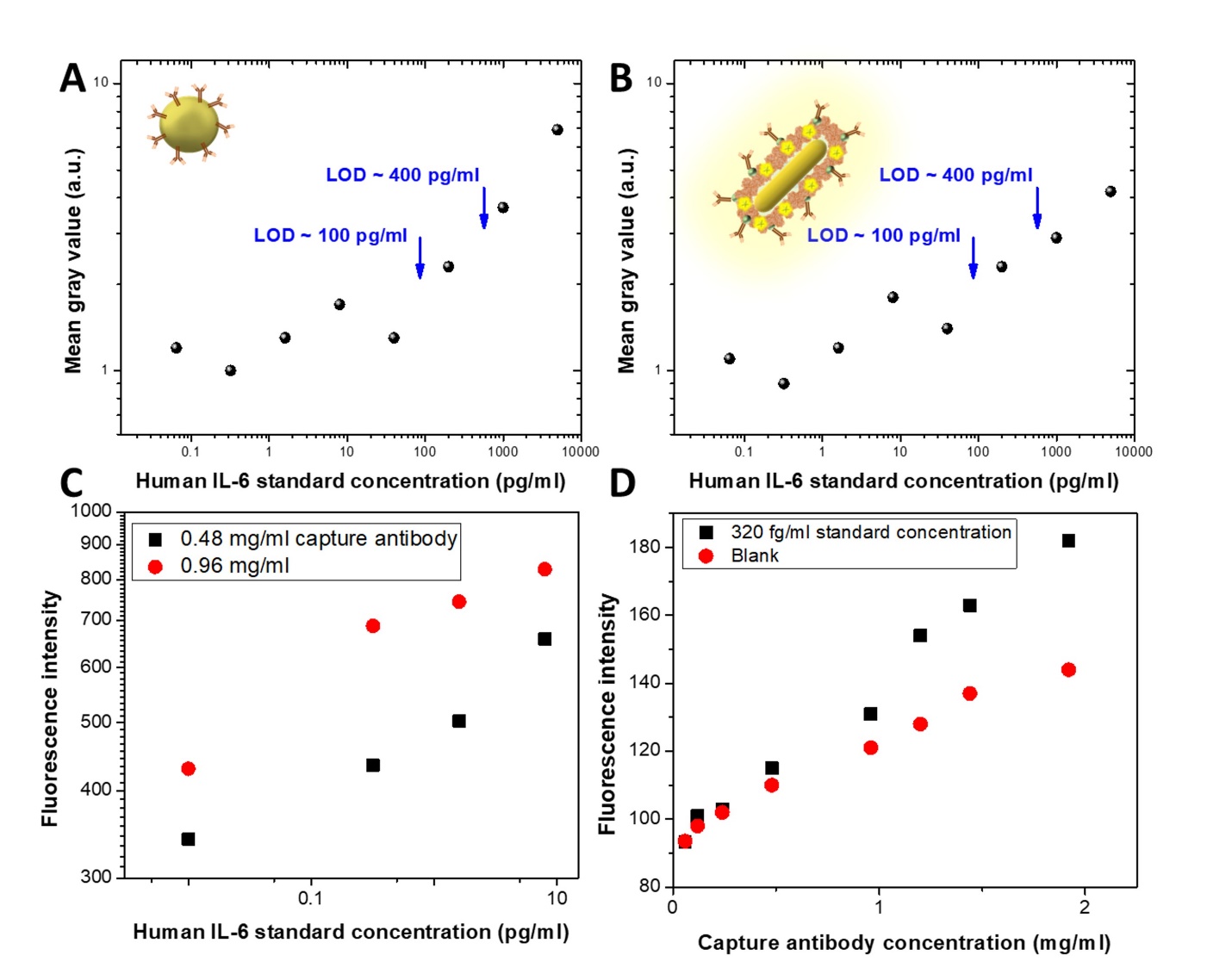


Figure S12**:** Dose-dependent mean gray values obtained from nitrocellulose membrane corresponding to different concentrations of IL-6 standards acquired from (A) AuNP-based and (B) plasmonic-fluor-based IL-6 LFA. (C) Dose-dependent mean gray values obtained from nitrocellulose membrane corresponding to different concentrations of IL-6 standards acquired from IL-6 p-LFA with different capture antibody concentrations. (D) Fluorescence intensity as function of capture antibody concentration.


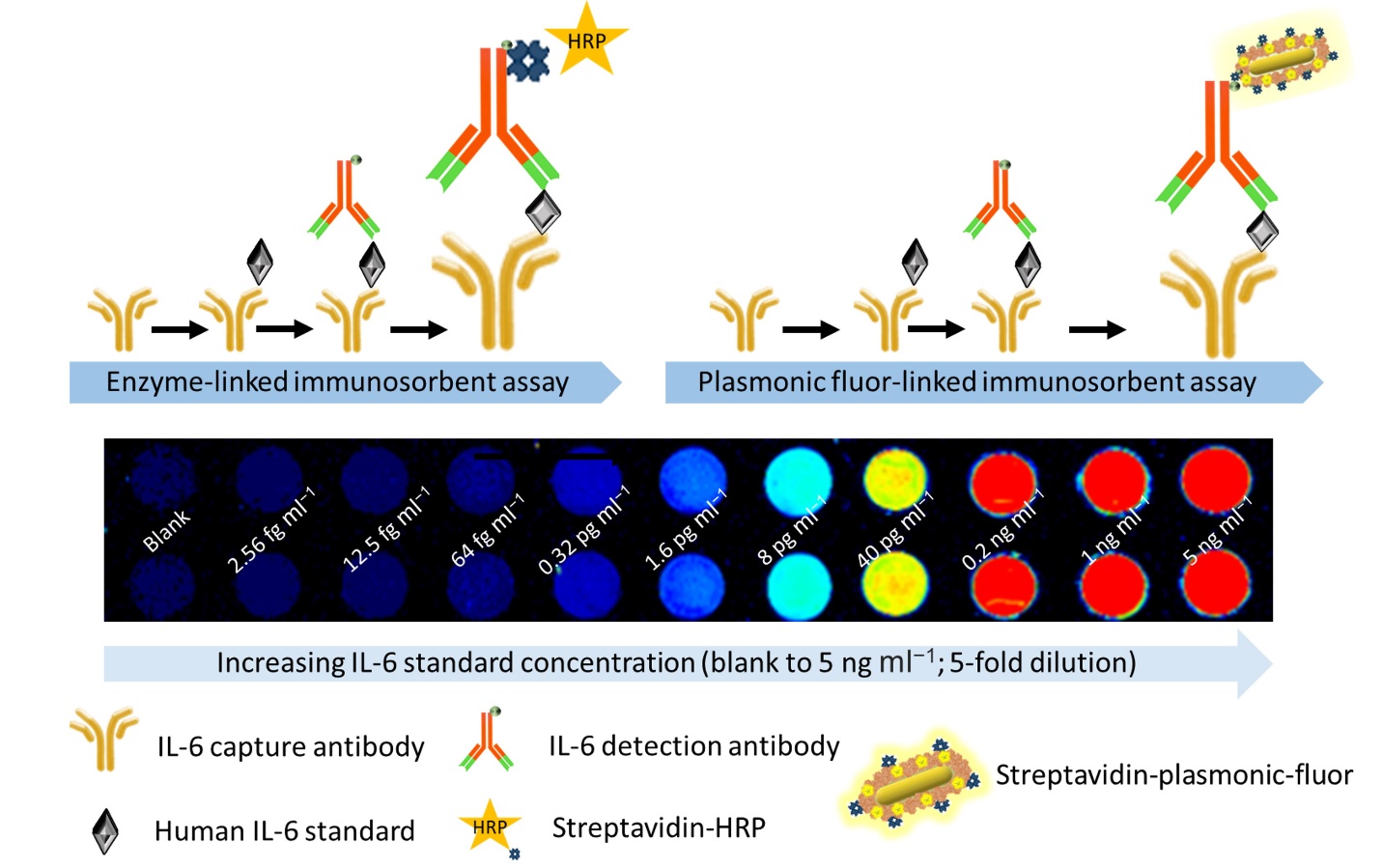


Figure S13**:** Schematic representation of (top left) ELISA and (top right) p-FLISA. (Bottom) Dose-dependent fluorescence intensity maps acquired by p-FLISA for different IL-6 concentrations.


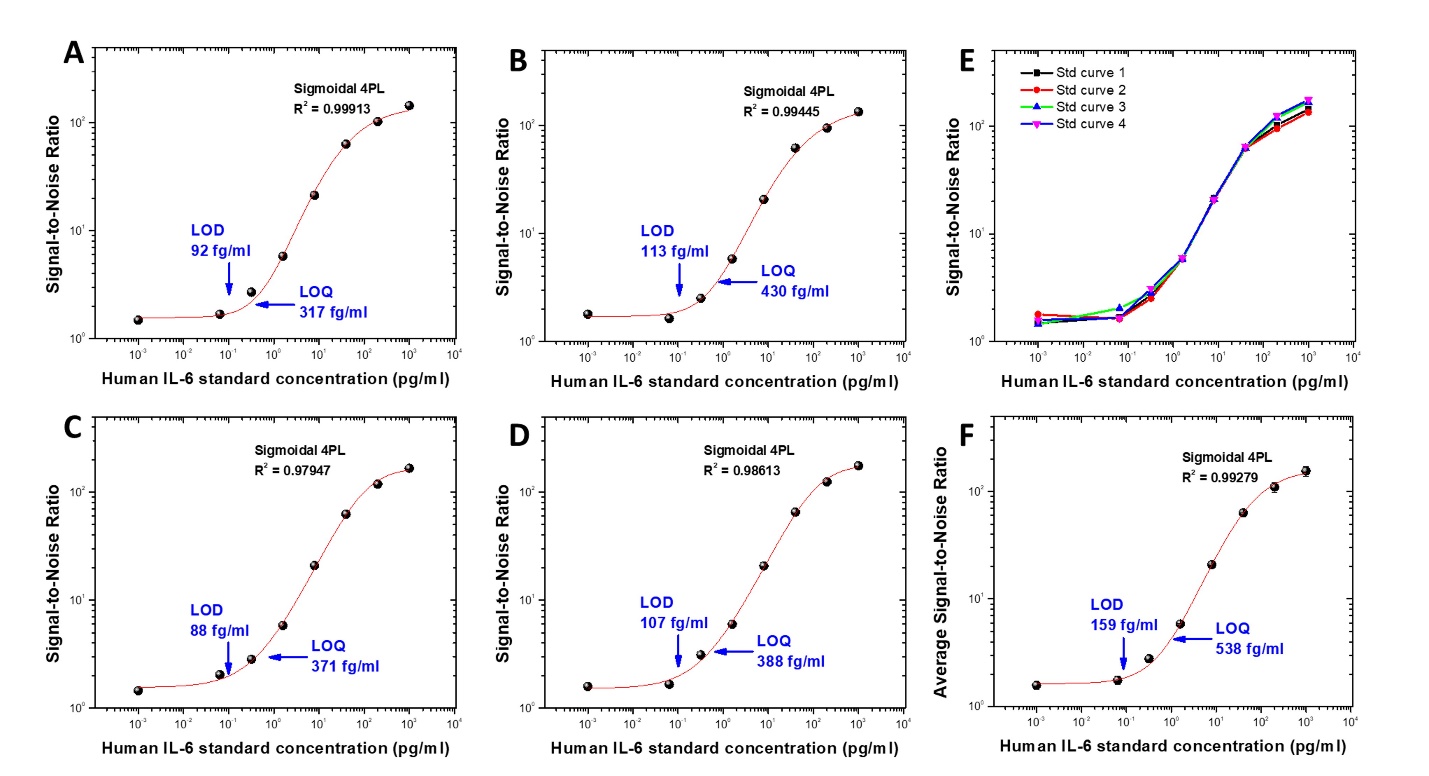


Figure S14**:** (A-D) Dose-dependent signal-to-noise ratio of multiple IL-6 p-LFA standard curves. (E) Overlaid dose-dependent signal-to-noise ratio of different standard curves depicting minimal deviation from one another. (F) Dose-dependent signal-to-noise ratio obtained by taking the average values from all four standard curves and its bioanalytical parameters. Error bars in figure F represent standard deviations from 8 different samples.


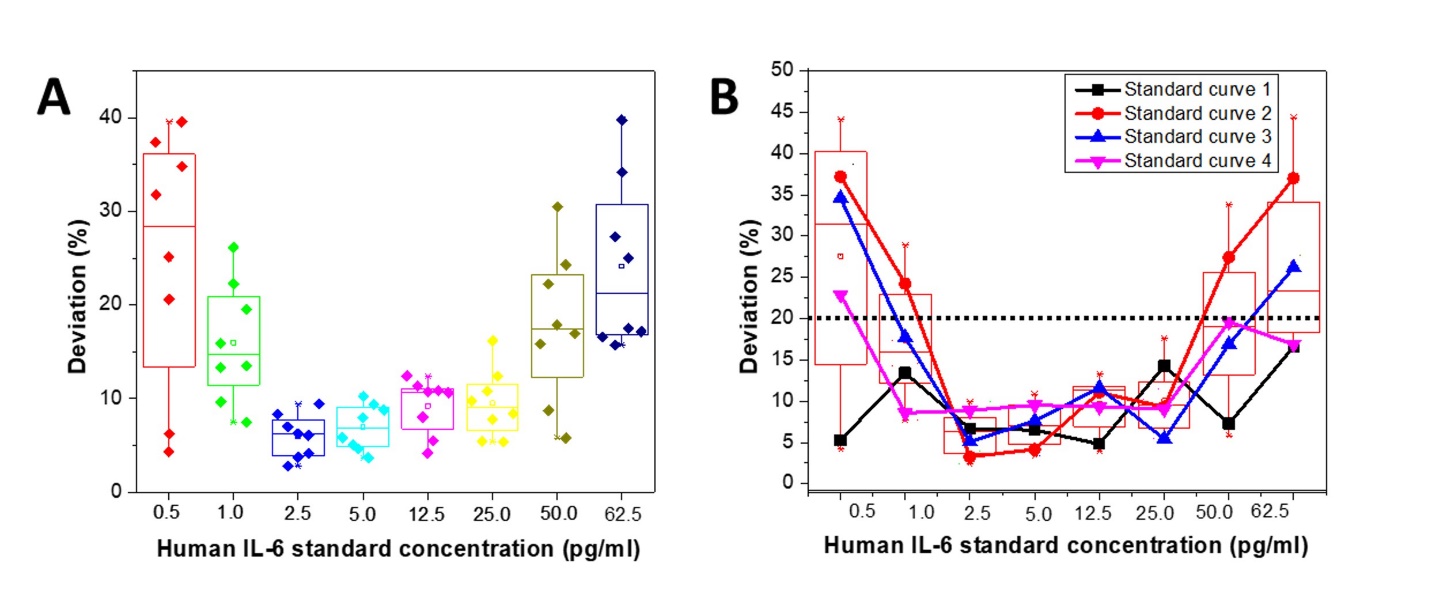


Figure S15**:** Multiple IL-6 standard curves were generated and samples with varying IL-6 concentrations (0.5 pg ml^−^1 to 62.5 pg ml^−1^) were tested in standard-free manner. Their experimental concentrations were determined using each standard curve, and deviation from actual concentration was calculated. (A) Samples were tested in duplicates in standard-free manner and for each test, experimental values were determined using 4 standard curves, thus, 8 data points corresponding to each concentration in the box plot. (B) Deviation in the average experimental values of the samples corresponding to each standard curve.


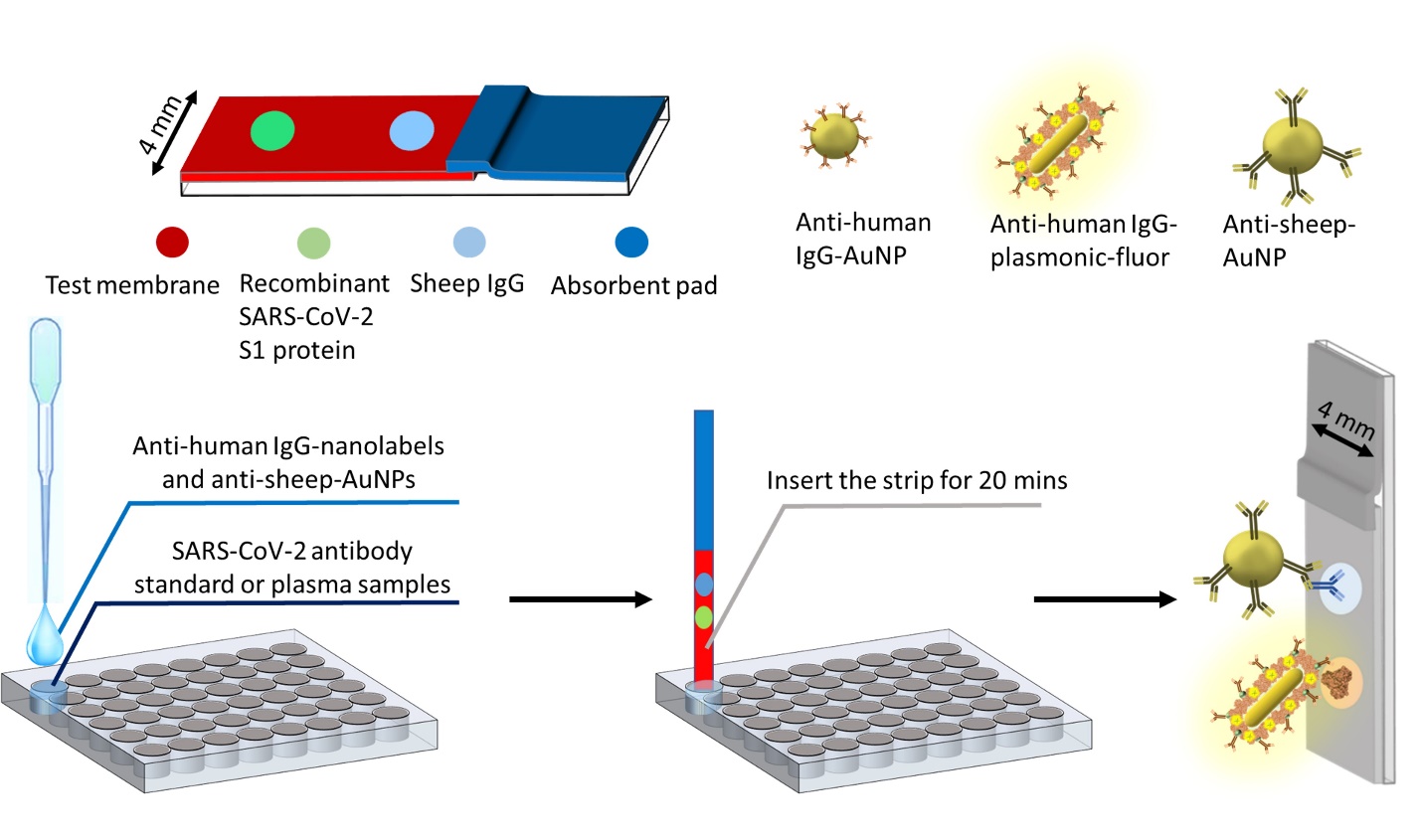


Figure S16**:** Schematic representation of SARS-CoV-2 S1 antibody lateral flow assay.


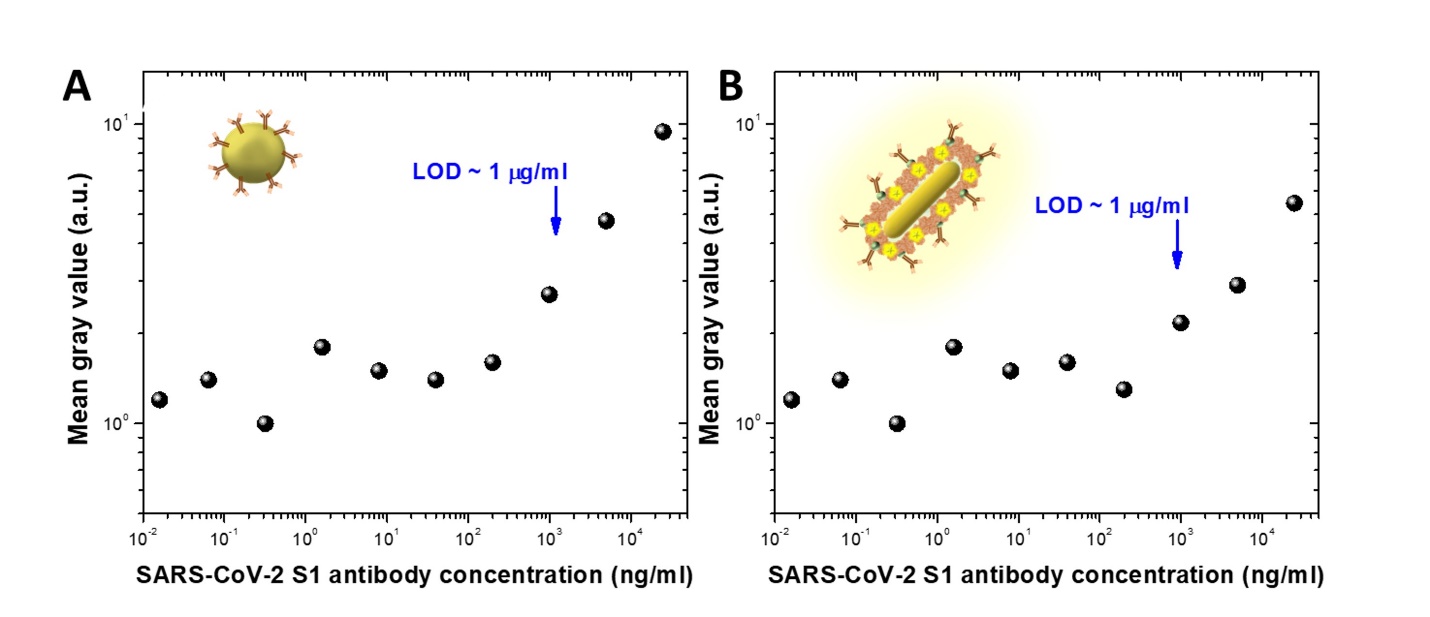


Figure S17**:** Dose-dependent mean gray values obtained from nitrocellulose membrane corresponding to different concentrations of SARS-CoV-2 S1 antibody solutions acquired from (A) AuNP-based and (B) plasmonic-fluor-based SARS-CoV-2 antibody LFA.


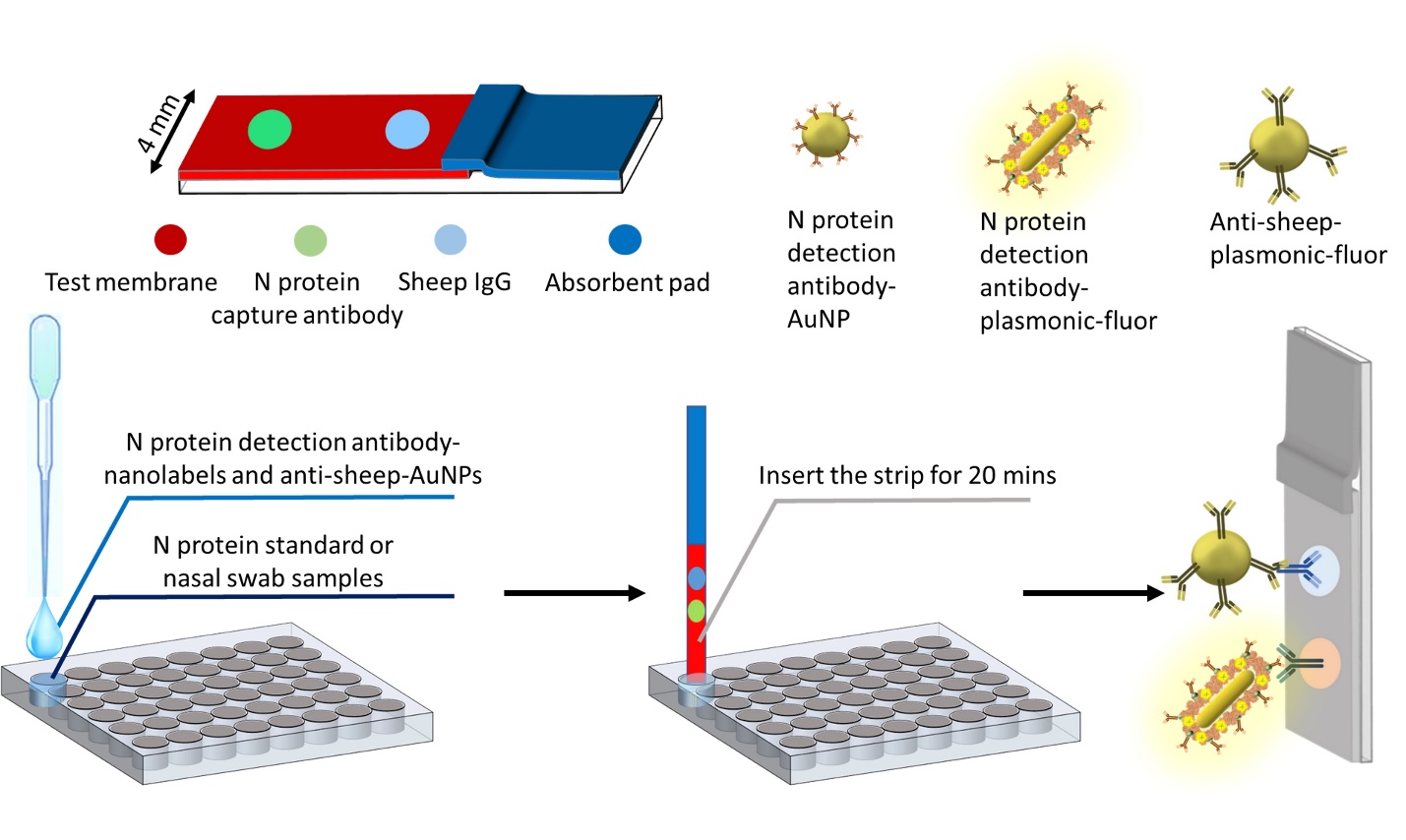


Figure S18**:** Schematic representation of nucleocapsid protein lateral flow assay.


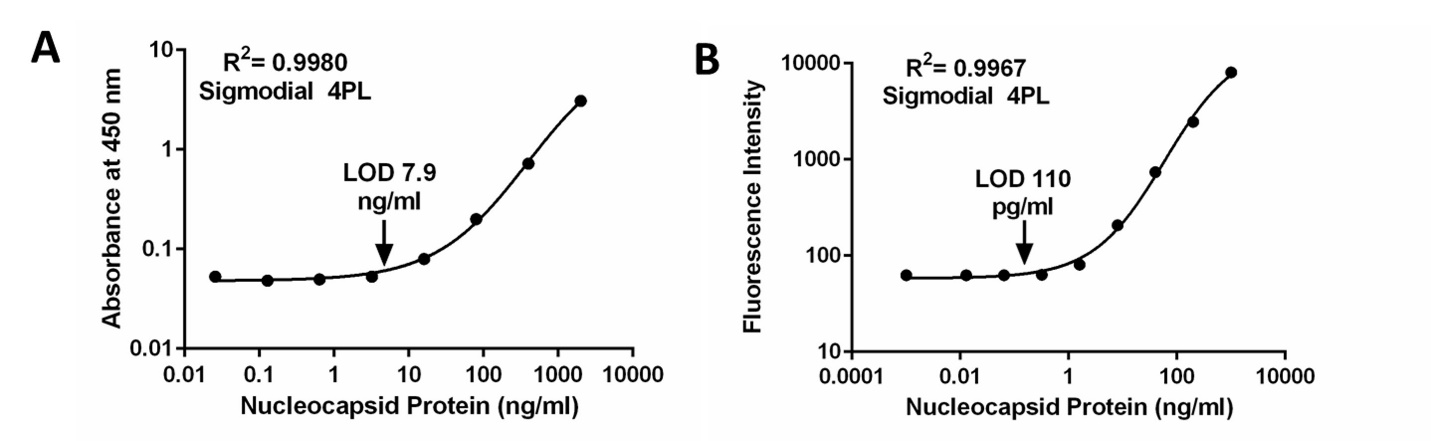


Figure S19**:** (A) Dose-dependent optical density corresponding to different concentrations of N protein in ELISA. (B) Dose-dependent fluorescence intensity corresponding to different concentrations of N protein in plasmonic-fluor linked immunosorbent assay (p-FLISA). Both the assays were implemented on microtiter plate and the sample-to-answer time was 4 h. Error bars represent standard deviations from two different samples.


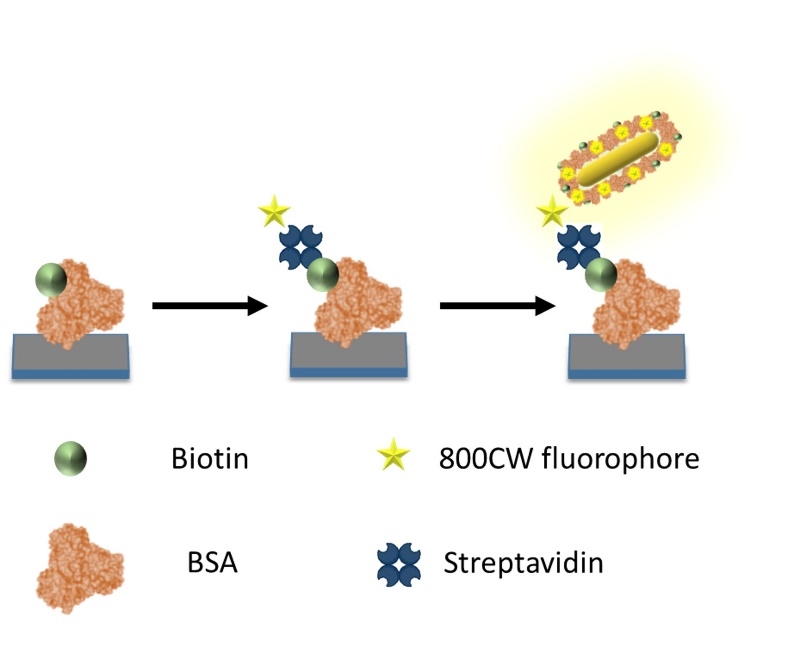


Figure S20. Schematic illustration of experiment designed to understand the enhancement of fluorescence signal using plasmonic-fluors compared to conventional fluorophores.

Table 1**:** Details of the patient samples tested using commercial antigen test (BD veritor^TM^) and digital pFLISA.

| Sample # | Age | Comorbidities* | CT  value | p-LFA result | Commercial Antigen Result | Duration of symptoms  (days) | Symptoms | Radiographic findings at diagnosis/testing: Chest X-ray (CXR) or Computed Tomography (CT) imaging | Severity/Critical illness/ Intensive care requirement |
| --- | --- | --- | --- | --- | --- | --- | --- | --- | --- |
| S1 | 33 | Asthma | 40.7 | Positive | Negative | 4 | Chest pain and constipation- nonspecific symptoms. | clear lungs | nil |
| S2 | 57 | none | 19.2 | Positive | Positive | 7 | Shortness of breath, Hypoxic Treated with remdesivir | Bilateral (b/l) interstitial infiltrates s/o viral pneumonia | nil |
| S3 | 49 | none | 27.9 | Positive | Negative | 14 | Flu like symptoms 2 weeks ago. | CT chest : Interstitial thickening and parenchymal infiltrates | nil |
| S4 | 59 | none | 30.5 | Negative | Negative | 3 | Cough malaise, Shortness of breath x 3 days, Hypoxia+ | b/l lower lobe infiltrates. | ICU admit for seizure unrelated to COVID |
| S5 | 62 | COPD | 42.8 | Positive | Negative | 2 | Shortness of breath. | clear lungs | nil |
| S6 | 77 | HTN, CKD | 22.6 | Positive | Positive | 1 | Shortness of breath. | b/l lower lobe infiltrates | Non – invasive ventilation, ICU admit |
| S7 | 27 | Psychosis | 39.5 | Positive | Negative | asymptomatic | None. COVID PCR + 2 weeks prior | clear lungs | nil |
| S8 | 55 | HIV (poorly controlled) | 21.8 | Positive | Positive | 14 | Cough, Shortness of breath 2 weeks. Hypoxia+ | left lower pneumonia | nil |
| S9 | 53 | ESRD, DM | 18.2 | Positive | Positive | 2 | Shortness of breath, Fever, Loss of taste. | b/l diffuse interstitial opacity | nil |
| S10 | 37 | none | 36.3 | Positive | Negative | asymptomatic | Presurgical screening. Tibial fracture | clear lungs | nil |
| S11 | 71 | DM | 18.8 | Positive | Positive | 3 | Loss of taste, Malaise, Shortness of breath | Patchy consolidation b/l | nil |
| S12 | 41 | DM | 39.5 | Positive | Negative | 3 | Chest pain- diagnosed as costochondritis. | clear lungs | nil |
| S13 | 58 | DM | 20.7 | Positive | Positive | 3 | Flu like illness, fever, myalgia | clear lungs | nil |
| S14 | 69 | DM, CAD | 23.8 | Positive | Negative | 3-5 | Few days flu like symptoms, | b/ll diffuse  interstitial opacities | ICU admit  Ventilated for hypoxic respiratory failure. Died |
| S15 | 44 | none | 32.7 | Positive | Negative | 14 | Cough, Shortness of breath for 2 weeks | clear lungs | nil |
| S16 | 70 | none | 29.3 | Positive | Negative | 2 | Fever, Cough, Shortness of breath | b/l mild  interstitial opacities | nil |
| S17 | 61 | HTN | 27.8 | Positive | Positive | 6 | Cough, Shortness of breath | b/l  interstitial and airspace opacities | nil |
| S18 | 42 | Obesity  CHF | 23.5 | Positive | Positive | 2 | Shortness of breath, cough, diarrhea, and myalgias | Multifocal PNA | ICU admit. Increase from home vent settings |
| S19 | 47 | COPD | 42.3 | Positive | Negative | 7 | Shortness of breath. | clear lungs | nil |

*DM= diabetes mellitus, COPD= chronic obstructive pulmonary disease, ESRD = End stage renal disease, HIV= human immunodeficiency virus infected, CHF= congestive heart failure, CAD coronary heart disease

**References:**

1. Luan, J.; Seth, A.; Gupta, R.; Wang, Z.; Rathi, P.; Cao, S.; Derami, H. G.; Tang, R.; Xu, B.; Achilefu, S., Ultrabright fluorescent nanoscale labels for the femtomolar detection of analytes with standard bioassays. *Nature biomedical engineering* **2020,** *4* (5), 518-530.

2. Gupta, R.; Luan, J.; Chakrabartty, S.; Scheller, E. L.; Morrissey, J.; Singamaneni, S., Refreshable Nanobiosensor Based on Organosilica Encapsulation of Biorecognition Elements. *ACS applied materials & interfaces* **2020,** *12* (5), 5420-5428.

3. Tebbe, M.; Kuttner, C.; Männel, M.; Fery, A.; Chanana, M., Colloidally stable and surfactant-free protein-coated gold nanorods in biological media. *ACS applied materials & interfaces* **2015,** *7* (10), 5984-5991.
